# From microbubbles to macro-control: Linking ultrasound-induced microarchitecture to precise drug release kinetics from acoustically responsive scaffolds

**DOI:** 10.1016/j.ultsonch.2026.107965

**Published:** 2026-07-22

**Authors:** Haijun Xiao, Jinye Xie, Somnath Maji, Mitra Aliabouzar, Mario L. Fabiilli

**Affiliations:** a Department of Radiology, University of Michigan, Ann Arbor, 48109, MI, USA; b Department of Biomedical Engineering, University of Michigan, Ann Arbor, 48109, MI, USA; c Department of Mechanical Engineering, University of Michigan, Ann Arbor, 48109, MI, USA; d Applied Physics Program, University of Michigan, Ann Arbor, 48109, MI, USA

**Keywords:** Ultrasound, Release kinetics, Phase-change droplets, Hydrogel, Fibrin, Acoustic droplet vaporization

## Abstract

Spatiotemporally controlled drug delivery systems are pivotal for advancing personalized medicine, yet programming precise release kinetics within implantable materials remains a significant challenge. Acoustically responsive scaffolds (ARSs), which utilize ultrasound to trigger payload release from phase-change emulsions embedded within a hydrogel matrix, offer a promising solution. However, the complex interplay of three ultrasound-based effects within an ARS – drug release kinetics, bubble morphology, and matrix mechanics – remains poorly understood. Here, we utilized a $2^{4}$ full factorial design to evaluate the effects of acoustic pressure, scanning velocity, step size, and fibrin concentration on drug release kinetics, rheological properties, and bubble morphology. This design enabled simultaneous estimation of parameter prioritization and interactions across the ARS process–structure–function response space. Using optimized linear models for twelve response variables, we show that release kinetics and rheological properties were orthogonally governed by acoustic pressure and fibrin concentration, respectively. The number of bubbles within the ARS, an indicator of acoustic droplet vaporization (ADV), correlated directly with acoustic pressure and fibrin concentration, which is consistent with trends of ADV efficiency and bubble coalescence, respectively. At high pressure, release correlated inversely with bubble-derived metrics (i.e., count, cross-sectional area, total surface area), which is consistent with a barrier-like effect of bubbles on drug diffusion. Mechanical properties (i.e., storage/loss moduli) and release kinetics exhibited diverging trends with fibrin concentration, thus revealing the importance of initial matrix concentration. Ultimately, this work helps elucidate critical relationships between process parameters and response variables, providing a quantitative framework for the rational design of personalized therapeutics using ARSs.

## Graphical abstract

**Figure:**
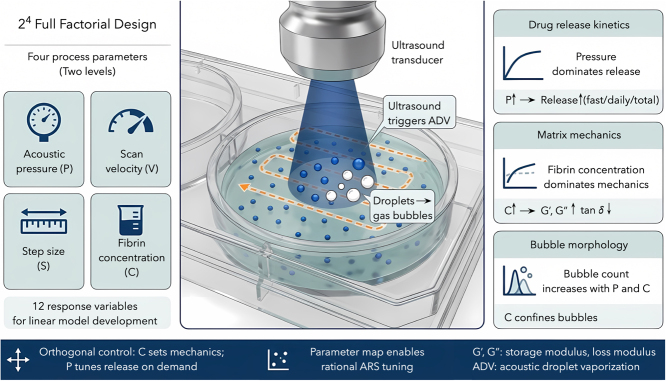

## 1 Introduction

Implantable hydrogels that release therapeutic agents in response to external stimuli [1] offer a route toward spatiotemporally controlled drug delivery, a long-standing goal in regenerative medicine and personalized therapeutics. Among candidate triggers, ultrasound is particularly advantageous: it penetrates deep tissue noninvasively, can be focused with millimeter-scale spatial precision, and is already integrated into clinical workflows [2], [3]. Recent delivery platforms have used preformed microbubbles embedded within hydrogels and CO${}_{2}$-loaded liposomes to promote release through bubble vibration, cavitation, or acoustic streaming [4], [5]. Acoustically responsive scaffolds (ARSs) exploit this ultrasound-responsive capability by embedding phase-change double emulsions within hydrogel matrices like fibrin [6], type I collagen [7], or alginate [8]. The double emulsion morphology of water-in-oil-in-water (W${}_{1}$/O/W${}_{2}$) enables encapsulation of hydrophilic therapeutic agents within the W${}_{1}$ phase. The oil phase is a hydrophobic perfluorocarbon liquid that minimizes outward diffusion of the encapsulated therapeutic agent. In the absence of ultrasound exposure, the emulsion within the ARS remains as stable, liquid droplets. When insonated above a threshold rarefactional pressure, the perfluorocarbon liquid within the emulsion droplets is non-thermally converted into a gas, a process termed acoustic droplet vaporization (ADV). Use of focused ultrasound enables spatially patterned bubble generation within an ARS post fabrication. ADV is being explored in many diagnostic and therapeutic applications [9], [10], [11], [12], [13], [14] ranging from contrast-enhanced imaging to targeted drug delivery. For ARSs, generation of gas bubbles and disruption of the double emulsion morphology by ultrasound simultaneously alters mass transport, scaffold microstructure, and local mechanics [6], [15]. This mechanism has been harnessed for on-demand growth factor release to promote angiogenesis [16], generation of microporous architectures that direct cell infiltration [17], and mechanical actuation of osteogenic differentiation through bubble-induced matrix remodeling [7]. The capacity to modulate both drug delivery and structural function within a single implant positions ARSs as a versatile therapeutic platform.

Substantial progress has been made in characterizing the physics of ADV and ARSs. ADV thresholds have been mapped as functions of ultrasound frequency, pulse repetition frequency, hydrogel density, and emulsion volume fraction [15], [18], [19], [20]. Ultra-high speed microscopy in combination with modeling has been used to identify the mechanisms governing ADV [21], [22], [23], [24]. ADV-induced changes to the hydrogel matrix, including spatially heterogeneous stiffening, fiber reorganization, and time-dependent relaxation, have been documented by atomic force microscopy and confocal imaging [25]. The relationship between acoustic parameters, droplet characteristics, and micropore geometry has been quantified, demonstrating that pore length scales with pressure, frequency, and droplet size while being constrained by matrix stiffness [17]. Most recently, visualization of bubble growth and drug front propagation has linked emergent multiphase morphology to sustained release kinetics [26], [27].

However, prior studies have predominantly employed low-dimensional parameter sweeps, varying one or two factors while holding others fixed. Three interconnected knowledge gaps therefore remain. First, the joint effects of acoustic, emulsion, and matrix parameters have not been systematically characterized; thus, the high-dimensional parameter space that governs ARS performance lacks a quantitative map. Second, although bubbles are central to ARS function, their mechanistic role in macro-scale drug release remains coarsely defined. Specifically, it remains unclear whether bubbles, which are indicators of vaporization events, are active drivers of convective transport and/or passive structural features that reshape diffusive pathways; and whether this role changes with process conditions [26], [28]. Third, matrix structure and mechanics are important, particularly as it impacts bubble dynamics during ADV [29] as well as subsequent drug diffusion [30] and cellular processes [31], but remain under-parameterized. It is not well understood how network density modulates the coupling between ADV events, mechanical evolution, and transport properties [17], [25].

Here we address these gaps through a $2^{4}$ full factorial experiment varying the following process parameters (i.e., independent variables): acoustic pressure, ultrasound transducer scanning velocity, scanning step size, and fibrin concentration. Twelve response (i.e., dependent) variables were monitored across three functional categories: drug release kinetics, rheological properties, and bubble morphology. A full factorial design was selected to ensure all main effects and interactions could be simultaneously estimated without confounding. Fibrin was selected as the hydrogel component of the ARS due to its widespread use within tissue engineering [32]. We developed optimized linear models for each response variable that included both main effects and interaction terms of all four independent variables. Using these models, we assessed the effect of terms using analyses of variance contribution and standardized coefficients. The most critical model terms were identified for each response variable *via* quadrant-based prioritization, thus enabling us to determine whether outcomes were governed by distinct or shared process parameters. A condition-stratified correlation analysis was then conducted to isolate associations between response variables of ARSs.

## 2 Materials and methods

### 2.1 Materials

Perfluoroheptane (C_7_F_16_, bulk boiling point: 80-82 °C) was acquired from Strem Chemicals (MA, USA). Alexa Fluor 488-labeled dextran (molecular weight 10 kDa), FluoroBrite Dulbecco’s modified Eagle’s medium (DMEM), and phosphate-buffered saline (PBS) were sourced from Life Technologies (NY, USA). Bovine fibrinogen, Pluronic F68, and bovine lung aprotinin were obtained from Sigma-Aldrich (MO, USA). Thrombin was procured from King Pharmaceuticals (TN, USA). Krytox 157 FSH originated from DuPont (DE, USA). Poly(ethylene glycol) bis(amine) (PEG, molecular weight 1000 g/mol) was purchased from Alfa Aesar (MA, USA).

### 2.2 Methods

#### 2.2.1 Experimental design

The study followed a $2^{4}$ full factorial design to evaluate the effects of four process parameters on ARS performance. Parameter levels (Table 1) were selected based on their use in prior studies [26], [27]. Each of the 16 combinations (Supplementary Table S1) was executed in triplicate, yielding 48 experimental runs. The eight combinations of acoustic pressure, scanning velocity, and step size are denoted by labels A–H (Supplementary Table S1). Twelve response variables spanning three categories were measured. Drug release kinetics were characterized by fast release, average daily release, and total release. Fast and total release denote the cumulative percentage of drug released at day 1 and 12, respectively, while average daily release is the mean incremental release percentage across days 2 through 12. Rheological properties included storage modulus ($G^{\prime }$), loss modulus ($G^{\prime \prime }$), and loss tangent (tanδ). Bubble-derived metrics characterized the number/area (i.e., bubble count, cross-sectional bubble area, individual bubble area, surface total bubble area) and shape (i.e., circularity, solidity).

**Table 1:** Process parameters and their levels for the $2^{4}$ full factorial design.

Process parameter	Low level	High level	Unit
Peak Negative Pressure	2.9	3.9	MPa
Scanning Velocity	3.0	6.0	mm/s
Scanning Step Size	1.3	2.6	mm
Fibrin Concentration	10.0	40.0	mg/mL

#### 2.2.2 Emulsion preparation

Double emulsions encapsulating Alexa Fluor 488-labeled dextran, a surrogate drug molecule, were prepared using microfluidics. Primary water-in-oil emulsions (W${}_{1}$/O) were formed by sonicating the aqueous dextran phase (1.67 mg/mL in PBS) with perfluoroheptane (C${}_{7}$F_16_) containing 2% (w/w) fluorosurfactant using a Q55 sonicator (QSonica, CT, USA). The W${}_{1}$/O ratio of the resulting primary emulsion was 1:2 (v/v). Double emulsions were generated on a glass microfluidic chip with a flow focusing junction (Part number 3200136, 14 x 17μm junction size, Dolomite, Royston, UK) with 50 mg/mL Pluronic F68 in PBS as the W${}_{2}$ phase. The primary emulsion and W${}_{2}$ phase were pumped at 0.5 μL/min and 2.5 μL/min, respectively, yielding droplets with nominal diameters of 13±2.8μm. Emulsions were sized using a Multisizer 4 Coulter counter (Beckman Coulter, CA, USA) and stored at 4 °C until use.

#### 2.2.3 Gel preparation

ARSs were prepared by combining bovine fibrinogen dissolved in degassed DMEM with bovine thrombin (2 U/mL), aprotinin (0.1 U/mL), and double emulsion (0.5% v/v). Listed compositions were the final concentrations in the gel. This volume fraction of emulsion was selected based its relevance in prior drug delivery studies [26], [27]. Fibrin concentration was set according to the experimental design (Table 1). Gels (volume = 0.3 mL) were polymerized in 24-well BioFlex plates (Flexcell International, NC, USA) for 15 min at room temperature, then covered with 0.5 mL DMEM supplemented with 100 U/mL penicillin and 100μg/mL streptomycin, and maintained in a standard tissue culture incubator at 37 °C with 5% CO${}_{2}$ throughout the experiment unless otherwise noted. Representative confocal micrographs of ARSs before and after ultrasound exposure are provided in Supplementary Figure S1 to illustrate the morphology of the gel/emulsion.

#### 2.2.4 Ultrasound exposure setup

A calibrated, single-element transducer (1.1 MHz, H-101, diameter: 64 mm, radius of curvature: 63.2 mm, Sonic Concepts, WA, USA) was driven by pulsed waveforms from a dual-channel function generator (33500B, Agilent Technologies, CA, USA) and amplified by a gated radiofrequency amplifier (GA-2500 A, Ritec Inc, RI, USA). The transducer was calibrated under free-field conditions using a custom fiber-optic hydrophone with a fiber diameter of 105μm and an automated scanning system (AIMS III, Onda Corporation, Sunnyvale, CA, USA). A frequency of 1.1 MHz was selected due to its relevance in therapeutic ultrasound [33], which permits greater tissue penetration and lower tissue attenuation compared to higher frequencies, as well as use in prior ARS studies [17], [26]. Peak negative pressure was set according to the experimental design; pulse duration and pulse repetition frequency were fixed at 12μs and 100 Hz (duty cycle 0.12%). These pulsing conditions, which have been used in previous studies to generate ADV, were selected to prevent thermal effects. The BioFlex plate was immersed in a degassed 37 °C water tank, and the transducer was positioned beneath the plate using a three-axis system controlled by MATLAB (MathWorks, MA, USA), with the axial focus placed 1 mm above the well bottom *via* pulse-echo alignment. The transducer was rastered across each well at the scan velocity and step size specified by the design. During release experiments, only the bottom surface of each BioFlex plate contacted the water bath, and the ultrasound beam passed through the approximately 0.2-mm-thick silicone elastomer membrane at the well bottom before reaching the ARS. Based on the reported attenuation coefficient of this membrane (0.7 ± 0.2 dB/MHz/cm), attenuation at 1.1 MHz was negligible [34]. Each well contained 0.5 mL of overlying medium, corresponding to an approximately 3-mm fluid height above the scaffold. Reflections from the medium–air interface can produce localized standing-wave fields; however, previous ARS experiments using a 2.5-MHz transducer showed that standing-wave-induced pressure enhancement was most pronounced within approximately one acoustic wavelength of the interface [35]. At 1.1 MHz, the wavelength in the medium was approximately 1.3 mm, placing the scaffold more than two wavelengths from the fluid–air interface. Therefore, substantial standing-wave-induced pressure enhancement at the scaffold location was not expected.

#### 2.2.5 Drug release quantification

Media overlying each gel was sampled daily over a 12-day period by removing 200 μL and replacing the withdrawn volume with fresh media to maintain sink conditions. The concentration of Alexa Fluor 488-labeled dextran in the collected samples was quantified by fluorescence spectrophotometry (excitation 495 nm, emission 525 nm) using a SpectraMax M2e microplate reader (Molecular Devices) at 37 °C. Calibration curves were constructed from serial dilutions of the stock dextran solution, with background correction applied to all readings. Cumulative release at each timepoint was calculated by mass balance, accounting for both the measured concentration and the fraction of analyte removed at preceding sampling events (Supplementary Figure S2). Three summary metrics were derived from the cumulative release profiles: fast release (release at day 1), average daily release (mean daily increment between days 2 and 12), and total release (cumulative release at day 12).

#### 2.2.6 Rheological measurements

Oscillatory shear measurements were performed on day 12 on a Discovery HR10 rheometer (TA Instruments) equipped with an 8 mm sandblasted parallel plate geometry. Prior to each measurement, the geometry and plate were cleaned with ethanol and the instrument gap was zeroed. For each sample, the gap was adjusted to achieve an axial compression force of 0.15–0.20 N, ensuring consistent contact without gel damage. Amplitude sweeps were conducted at a fixed angular frequency of 1 Hz (6.28 rad/s) with oscillation torque logarithmically increasing from 0.1 to 900 μN⋅m. Storage modulus ($G^{\prime }$) and loss modulus ($G^{\prime \prime }$) at 1% strain were extracted by natural spline interpolation of the amplitude sweep data. Loss tangent was calculated as $\tan\delta =G^{\prime \prime }/G^{\prime }$. A strain of 1% was selected as a representative point within the linear viscoelastic region, where moduli remained independent of strain amplitude (Supplementary Figure S3).

#### 2.2.7 Optical characterization of surface bubble morphology

Surface bubble segmentation was performed using Cellpose-SAM (CPSAM) [36], the default model in Cellpose 4.0.8 that integrates a Segment Anything Model transformer backbone with instance segmentation capabilities (Supplementary Figure S4). Shape descriptors were extracted from label masks using Fiji. Measured parameters included: (1) bubble count, representing total detected instances per image; (2) individual bubble area, the projected surface area of each bubble; (3) total area, the cumulative coverage of all bubbles; (4) circularity, defined as $4\pi \times \text{Area}/{\text{Perimeter}}^{2}$, quantifying deviation from a perfect circle; and (5) solidity, defined as Area/Convex Hull Area, detecting coalescence events that create concave boundaries. All area measurements were converted to physical units (${\text{mm}}^{2}$) using image scale calibration. Summary statistics including mean, median, geometric mean, and standard deviation were computed for area, circularity, and solidity. The median was adopted as the primary measure for statistical analysis given its robustness to outliers in skewed bubble size distributions.

#### 2.2.8 Cross-sectional bubble area

On day 12, B-mode images of the gel within the BioFlex plate were acquired using a Zonare ZS3 Ultrasound System (Mindray) and an L30-8 linear probe operating at 24 MHz. Imaging depth was set to 3.0 cm. Acoustic power was minimized to avoid near-field saturation while maintaining adequate signal intensity. Cross-sectional bubble area was quantified using a reflector projection algorithm (Supplementary Figure S5), in which the detected upper bubble boundary was vertically projected to estimate the bubble-affected cross-sectional area within a defined region of interest. Additional details regarding the algorithm are provided in the Supplementary Materials.

#### 2.2.9 Statistical analysis

Full factorial linear models were fit to each response variable after centering all process parameters, with the saturated model containing all 15 terms in the $2^{4}$ design (four main effects, six two-factor interactions, four three-factor interactions, and one four-factor interaction). For each response variable, three candidate models were considered: a saturated model; a stepwise-reduced model based on Akaike information criterion (AIC) optimization; and a DHARMa-guided [37] reduced model that retained all four main effects and added only higher-order terms identified as significant by either Type III analysis of variance or robust Wald testing, depending on the preliminary residual diagnostic outcome. Models were selected using a composite score balancing fit, diagnostic validity, and parsimony, with adjusted $R^{2}\times 100$ as the base score and penalties assigned for failures of the DHARMa uniformity, dispersion, and outlier tests and for increasing parameter count. Preliminary DHARMa screening was used during model selection, and selected models were subsequently subjected to enhanced diagnostics. Residual assumptions were assessed *via* DHARMa [37] simulation-based diagnostics evaluating uniformity, dispersion, and outliers. Statistical inference employed Type III analysis of variance (ANOVA) with heteroscedasticity-consistent standard errors (HC3) when diagnostic tests indicated non-constant variance or other residual-assumption violations; otherwise, standard Type III ANOVA was applied. All p-values were false discovery rate (FDR)-adjusted using the Benjamini–Hochberg procedure [38] ($p_{\text{FDR}}<0.05$). Effect importance was evaluated by variance contribution and standardized coefficients ($\beta^{*}$), where variance contribution was calculated from the Type III sum of squares for each effect relative to the total model-explained sum of squares. Directional consistency of significant effects was evaluated by computing marginal effects across process conditions; effects were classified as robust, moderated, or reversed based on confidence interval patterns across the experimental space. A quadrant framework integrating statistical significance with variance contribution prioritized process parameters, and model performance was further summarized using 10-fold cross-validated root mean square error (RMSE) and variance inflation factors. Response correlations were stratified by pressure and concentration. Full statistical procedures are detailed in the Supplementary Methods.

## 3 Results

### 3.1 Model development and evaluation

For each of the twelve response variables, the three candidate models were screened to select the best model for use in subsequent analyses. DHARMa-optimized models were selected for seven responses and stepwise-reduced models for the remaining five (Supplementary Figure S6). Adjusted $R^{2}$ values ranged from 0.29 (loss tangent) to 0.92 (storage modulus), with ten of twelve models exceeding 0.65. The comparatively low explanatory power for loss tangent reflects the ratio-derived nature of this variable ($\tan\delta =G^{\prime \prime }/G^{\prime }$), which represents a relative viscous-to-elastic balance derived from two parent moduli. A supplemental repeatability and residual-structure analysis was performed for the rheological responses (Supplementary Table S3). The median within-condition coefficient of variation for loss tangent was 18.0%, within the same range as storage and loss moduli (16.3% and 13.5%, respectively). Adding all higher-order process interactions did not significantly improve the loss tangent model relative to the main-effect model (p=0.291), and residual correlations with measured release, bubble, and rheological variables yielded no FDR-significant associations. These results identified loss tangent as the weakest rheological response in terms of process-parameter explanatory power, while storage and loss moduli remained the stronger rheological readouts of matrix mechanics. Cross-validated prediction errors (CV RMSE) scaled proportionally with response magnitude, ranging from 0.005 (solidity) to 25.5 (bubble count), with no evidence of overfitting in any selected model.

Eleven models satisfied all DHARMa diagnostic criteria (p>0.05); circularity failed the outlier test (p=0.019) and was analyzed using HC3 robust inference (Supplementary Figure S7). Variance inflation factors remained below 2 for all model terms, confirming negligible multicollinearity. Likelihood ratio tests confirmed that no selected reduced model significantly compromised explanatory power relative to the saturated model.

**Fig. 1:**
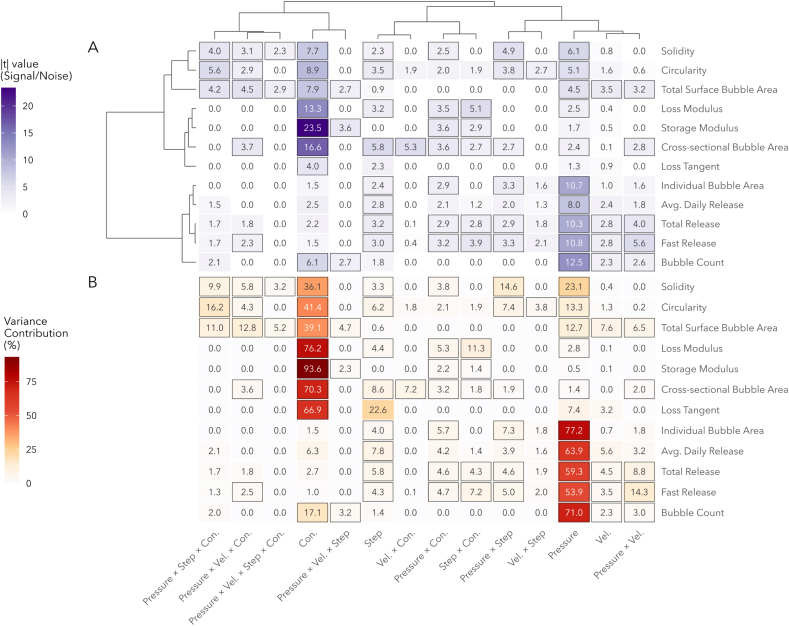
Dual-metric heatmaps of process parameter reliability and importance across response variables. (A) Absolute t values, indicative of statistical signal strength, are shown in a purple gradient. (B) Variance contribution, indicative of the relative importance within each response, is displayed in an orange gradient. Rows and columns were hierarchically clustered by profile similarity, not magnitude. In panel B, the sum across each row is 100. Black borders denote statistical significance based on FDR-correction (p<0.05). The process parameters of velocity (vel) and fibrin concentration (con) are listed in abbreviated formats. Process parameter values corresponding to each condition series are listed in Supplementary Table S1.

### 3.2 Process parameter reliability and variance contribution

Using the selected model for each response variable, we then identified which process parameters were most statistically reliable and accounted for the largest share of explained variance. Dual-metric heatmaps provide complementary perspectives on process parameter importance (Fig. 1). The |*t*|-value matrix displays statistical reliability across all effect–response combinations (Fig. 1A). The variance contribution matrix quantified each effect’s share of explained variance within each response, enabling cross-response comparison through percentage normalization (Fig. 1B). For example, the concentration effect on storage modulus showed a |*t*| value of 23.5 and accounted for 93.6% of the explained variance. As expected from their mathematical linkage, effects with stronger statistical signals consistently yielded higher variance contributions.

Hierarchical clustering of responses revealed two groups based on shared sensitivity patterns (Fig. 1A). Release kinetics (i.e., fast, daily, total) clustered with surface bubble count and median bubble area. Rheological properties (i.e., storage modulus, loss modulus, loss tangent) grouped with cross-sectional bubble area, surface total bubble area, and bubble shape parameters (i.e., circularity, solidity).

Column clustering partitioned effects into two primary groups (Fig. 1A). The first group contained concentration alongside third- and fourth-order interactions involving multiple parameters. The second group aggregated the main effects of pressure, velocity, and step size with all pairwise interactions, including those involving concentration.

Cross-referencing both panels (Fig. 1A,B) enabled identification of dominant factors satisfying both high reliability (|*t*|> 2) and variance contribution (> 10%). Justification for these thresholds are found in the Supplementary Materials. Concentration met these criteria for all rheological responses and cross-sectional bubble area. Pressure met these criteria for release metrics and surface bubble count. A separate category comprised effects achieving significance with modest variance contribution (< 10%), concentrated among pairwise interactions of ultrasound parameters.

**Fig. 2:**
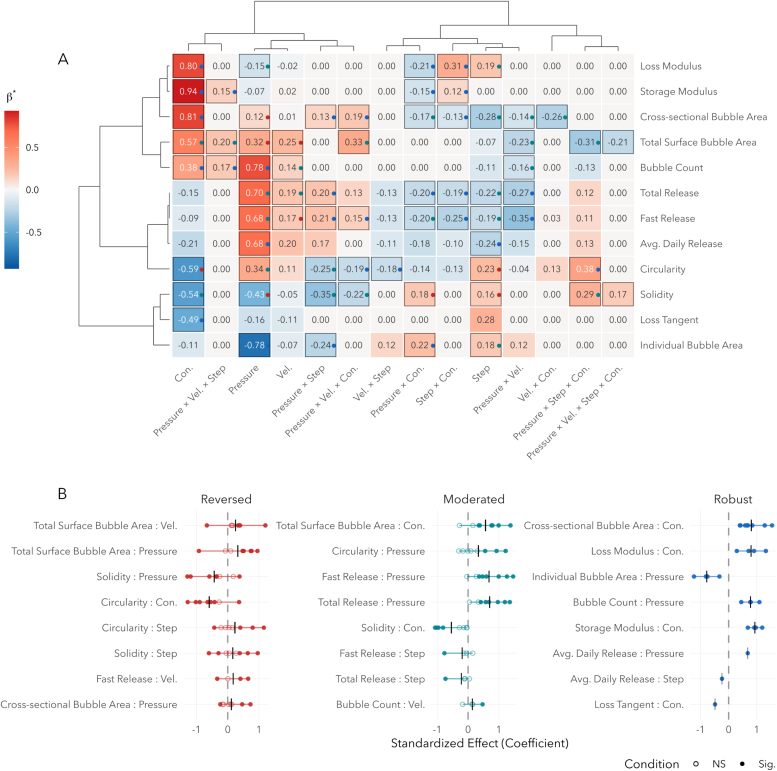
Standardized coefficients ($\beta^{*}$) and process parameter stability across response variables. (A) Standardized $\beta^{*}$ heatmap shows effect magnitude and direction (blue–white–orange diverging scale). Colored dots indicate directional consistency across three regimes: robust (blue, consistent across conditions), moderated (teal, strength varies), or reversed (red, direction changes). Both axes are clustered by profile correlation. Black borders denote statistical significance based on FDR-correction (p<0.05). (B) Representative main effects under varying moderator conditions were grouped by stability regime. Horizontal spans show how standardized effects shift across condition combinations; filled circles indicate significant, open circles non-significant. Vertical bars mark $\beta^{*}$ at centered values. Effects crossing zero confirm directional reversal. The process parameters of velocity (vel) and fibrin concentration (con) are listed in abbreviated formats. Process parameter values corresponding to each condition series are listed in Supplementary Table S1.

### 3.3 Process parameter direction and stability

Next, we assessed the magnitude, significance, and directionality of the correlations between process parameters and response variables; a focus of the analysis was whether significant effects remained directionally stable across different combinations of process parameters. The standardized coefficient ($\beta^{*}$) heatmap displays effect magnitude and direction, with diverging colors distinguishing positive from negative influences (Fig. 2A). Hierarchical clustering based on coefficient profiles maintained certain groupings from the |*t*|-value clustering while revealing divergences. Release metrics remained tightly grouped, as did rheological properties and main effects of pressure and velocity. Bubble morphology parameters exhibited greater positional variability. For example, individual bubble area moved toward loss tangent and solidity, circularity and solidity separated, and bubble count shifted away from release metrics.

Directional consistency of $\beta^{*}$ was assessed for all significant effects by examining conditional distributions across all combinations of process parameters (Fig. 2B). Robust effects maintained consistent direction (i.e., remained entirely positive or negative) and significance across all conditions. Moderated effects preserved direction but varied in magnitude, and thus were not significant across certain combinations. Reversed effects changed direction depending on levels of process parameters. These classifications were annotated as colored markers on the heatmap (Fig. 2A).

Most significant effects fell into robust or moderated categories. Concentration effects on rheological properties were uniformly robust with positive values for storage and loss moduli but negative for loss tangent. Pressure effects on release metrics remained positive, with daily release robust and fast and total release moderated.

Reversed effects were concentrated among bubble morphology parameters. Circularity reversed for concentration and step size while solidity reversed for pressure, step size, and the interaction of pressure with concentration. Surface total bubble area reversed for both pressure and velocity. Among release metrics, only velocity effects on fast release reversed, shifting from positive at 2.9 MPa to negative at 3.9 MPa.

**Fig. 3:**
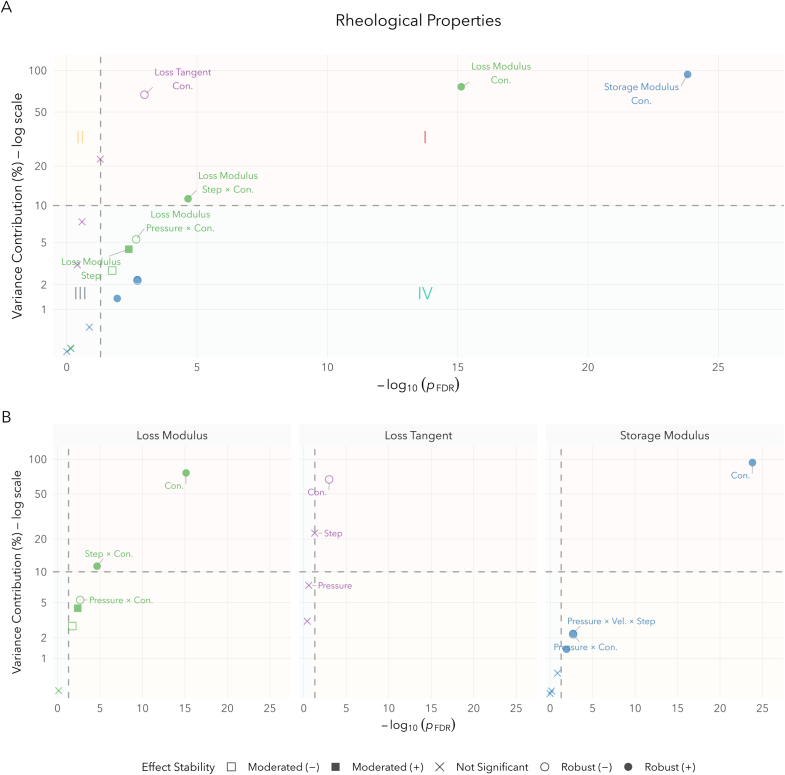
Process parameter prioritization for rheological properties. (A) A quadrant plot maps FDR-adjusted statistical significance ($-\log_{10}\left(p_{\text{FDR}}\right)$) against variance contribution (VC%), with VC% on a log scale. Vertical and horizontal thresholds denote $p_{\text{FDR}}=0.05$ and VC% = 10%, respectively. Quadrants follow Cartesian convention: I (significant, high VC%), II (non-significant, high VC%), III (non-significant, low VC%), and IV (significant, low VC%). Symbols indicate directional consistency across all process conditions. (B) Response-specific faceted views. Fibrin concentration dominated all three responses (quadrant I, robust), while other significant effects primarily populate quadrant IV (statistically significant but contributing little variance). The process parameters of velocity (vel) and fibrin concentration (con) are listed in abbreviated formats.

### 3.4 Regulatory architectures of system responses

#### 3.4.1 Process parameter prioritization for rheological properties

We then investigated which process parameters were the highest-priority regulators of rheological behavior. The quadrant plot integrated statistical reliability with variance contribution to prioritize process parameters for rheological responses (Fig. 3A). Effects occupying quadrant I (i.e., upper right) satisfied the dual criteria of high signal strength (i.e., > $-\log_{10}\left(p_{\text{FDR}}\right)$ where $p_{\text{FDR}}$ = 0.05) and substantial variance share (i.e., > 10% variance contribution).

Concentration was the most critical process parameter for all three rheological properties, with a robust positive influence on viscoelastic moduli and negative influence on loss tangent across all tested conditions (Fig. 3B). The negative effect on loss tangent revealed that storage modulus increased proportionally more than loss modulus across the tested range of fibrin concentration, physically reflecting a shift toward a more elastic, solid-like network architecture at higher fibrin densities. The interaction of step size with concentration was also critical for loss modulus. Remaining significant effects clustered in quadrant IV, exhibiting statistical reliability but limited variance contribution. These included interactions of pressure with concentration and the three-way interaction of pressure, velocity, and step size for storage modulus.

**Fig. 4:**
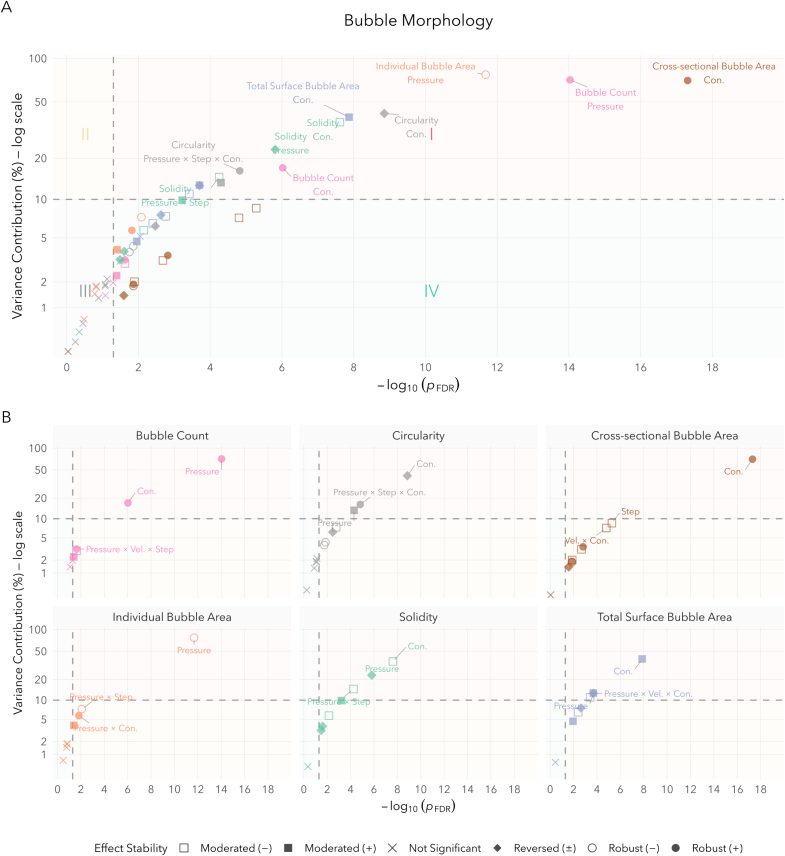
Process parameter prioritization for bubble metrics. (A) A quadrant plot maps FDR-adjusted statistical significance ($-\log_{10}\left(p_{\text{FDR}}\right)$) against variance contribution (VC%). Vertical and horizontal thresholds denoted $p_{\text{FDR}}=0.05$ and VC% = 10%, respectively. Symbols indicate directional consistency across all process conditions. (B) Response-specific faceted views. Concentration and pressure were dominant quadrant I factors for bubble area and count. For shape parameters (i.e., circularity, solidity), critical factors in quadrant I exhibited directional reversal or moderation, indicating dependency on interaction partners. The process parameters of velocity (vel) and fibrin concentration (con) are listed in abbreviated formats.

#### 3.4.2 Process parameter prioritization for bubble morphology

We then determined which parameters most strongly impacted bubble morphology. Quadrant analysis revealed a more complex control structure for bubble morphology than for rheological properties (Fig. 4A). Rather than a single factor, both concentration and pressure occupied quadrant I across multiple metrics. Bubble count was controlled by robust, primary and secondary factors of pressure and concentration, respectively (Fig. 4B). Both factors maintained a positive direction across all process conditions, enabling reliable prediction of bubble count from either factor independently. Both cross-sectional bubble area and median bubble area were dominated by a single process parameter; the former was dependent on fibrin concentration, which displayed a robust positive effect, while the latter on pressure, with a robust negative influence.

Shape parameters presented a fundamentally different pattern. Multiple effects were in quadrant I for circularity, solidity, and total surface bubble area, but these effects exhibited mixed stability profiles (Fig. 4A,B). For circularity, concentration contributed the largest variance share yet displayed directional reversal; pressure contributed less but maintained moderated positive behavior. For solidity, concentration showed a moderated negative direction while pressure exhibited reversal. Surface total bubble area followed a similar pattern with concentration exhibiting a moderated, positive direction and pressure reversing across conditions. Quadrant IV contained numerous significant effects with limited variance contribution, predominantly interactions involving pressure and velocity. No effects were in quadrant II, suggesting that high-variance effects generally achieved statistical reliability in this dataset.

**Fig. 5:**
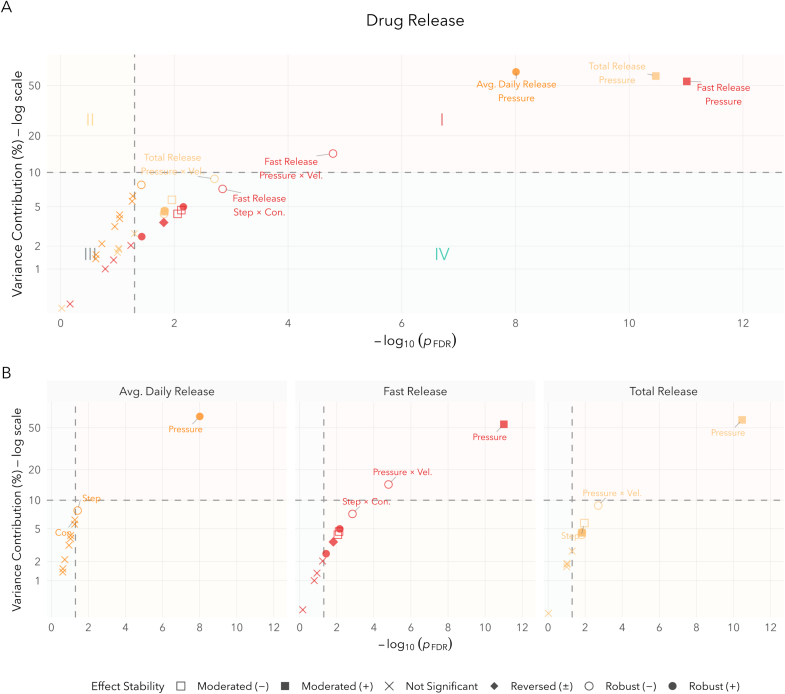
Process parameter prioritization for drug release kinetics. (A) A quadrant plot maps FDR-adjusted statistical significance ($-\log_{10}\left(p_{\text{FDR}}\right)$) against variance contribution (VC%). Vertical and horizontal thresholds denote $p_{\text{FDR}}=0.05$ and VC% = 10%, respectively. Symbols indicate directional consistency across all process conditions. (B) Faceted views by response variable. For all metrics of drug release kinetics, pressure was the most critical process parameter, while most higher-order interactions clustered in the bottom quadrants. The process parameters of velocity (vel) and fibrin concentration (con) are listed in abbreviated formats.

#### 3.4.3 Process parameter prioritization for drug release kinetics

Similarly, we evaluated which parameters were dominant regulators of drug release kinetics. Pressure was the most critical process parameter for all three metrics of drug release kinetics, with no directional reversal (Fig. 5B). Average daily release exhibited robust positive behavior, while fast and total release showed moderated positive effects with magnitude varying across experimental conditions. The interaction of pressure with velocity also reached quadrant I for fast release, with a robust negative direction.

Concentration, despite its dominant role in rheological properties, contributed minimally to release kinetics. Its main effects on all three release metrics fell below both reliability and variance thresholds, occupying quadrant III. This divergence underscores the complexity of the ARS with formulation-dependent and ultrasound-dependent responses. Quadrant IV contained multiple pairwise interactions involving pressure, velocity, and step size. These effects achieved statistical reliability but accounted for limited variance. Step size interactions with concentration showed reliable negative effects on fast and total release within this quadrant.

**Fig. 6:**
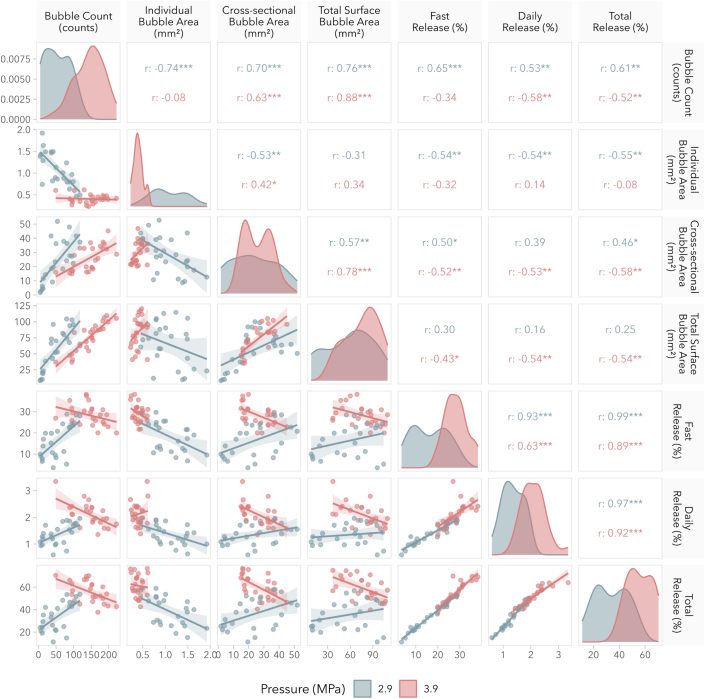
Paired correlation matrix of bubble and drug release metrics, grouped by acoustic pressure. The upper triangle of the matrix displays the Pearson correlation coefficients for each pressure level with significance indicators (* p<0.05, ** p<0.01, *** p<0.001). The lower triangle shows scatter plots with group-specific linear fits. For the upper and lower triangles, the column and row headings denote the metrics plotted on the x- and y-axes, respectively. Along the main diagonal, density distributions of data denoted by column heading are displayed. n = 24 per group.

### 3.5 Response correlations

We then evaluated how response variables were correlated across different pressures and fibrin concentrations. Correlation analyses grouped by acoustic pressure revealed pressure-dependent relationships among bubble characteristics and drug release metrics (Fig. 6). Because bubble morphology was measured only at the terminal timepoint, these analyses identify endpoint associations with release behavior rather than direct evidence of dynamic structure–function coupling throughout the 12-day release period. Bubble count correlated positively with all release metrics at low pressure. At high pressure, daily and total release reversed to significantly negative correlations, whereas fast release was not significantly correlated with bubble count. Bubble area showed significant negative correlations with all release metrics at low pressure, but these associations lost significance at high pressure. The strong negative correlation between bubble count and median area observed at low pressure also disappeared at high-pressure.

Cross-sectional and total surface bubble areas displayed distinct pressure-dependent patterns with release. At low pressure, cross-sectional area correlated positively with fast and total release but showed no significant association with daily release; total surface bubble area exhibited no significant correlations with any release metric. High pressure produced a uniform shift, with both bubble area metrics exhibiting significant negative correlations with all three release metrics.

Certain relationships remained stable across pressure conditions. Bubble count was positively correlated with both cross-sectional and total surface bubble areas regardless of pressure. Cross-sectional and total surface bubble areas also preserved their positive association. Similarly, release metrics exhibited strong positive intercorrelations at both pressures, with all pairwise associations remaining statistically significant despite modest attenuation at high pressure.

**Fig. 7:**
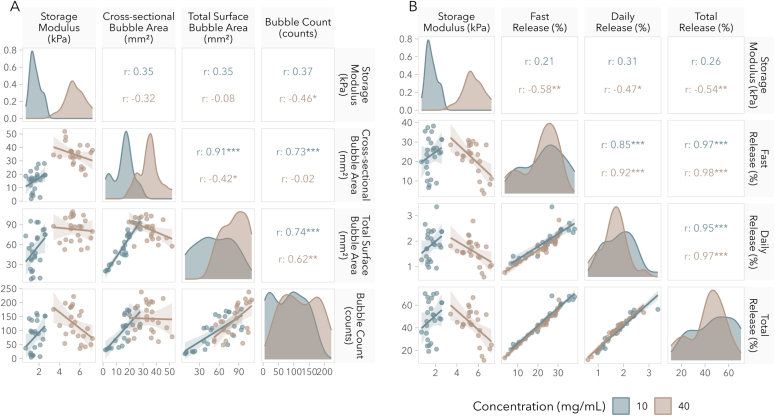
Concentration-dependent correlation patterns linking mechanics to structure and function. (A) Storage modulus versus bubble morphology metrics. (B) Storage modulus versus drug release metrics. In both panels, the upper triangle of the matrix displays the Pearson correlation coefficients for each fibrin concentration with significance indicators (* p<0.05, ** p<0.01, *** p<0.001). The lower triangle shows scatter plots with linear fits. For the upper and lower triangles, the column and row headings denote the metrics plotted on the x- and y-axes, respectively. Along the main diagonal, density distributions of data denoted by column heading are displayed. n = 24 per group.

Concentration-grouped analyses revealed distinct patterns between scaffold mechanics, bubble characteristics, and drug release (Fig. 7). At low concentration, storage modulus showed no significant correlations with any bubble metric (Fig. 7A). At high concentration, a significant negative correlation emerged between storage modulus and bubble count, while associations with cross-sectional and total surface bubble areas remained non-significant. Among bubble metrics, bubble count was positively correlated with total surface bubble area at both concentrations. However, the strong positive correlation between bubble count and cross-sectional area observed at low concentration disappeared at high concentration. A similar pattern was observed with cross-sectional and total surface bubble areas. Storage modulus was not significantly correlated with any release metric at low concentration (Fig. 7B). At high concentration, significant negative correlations emerged between storage modulus and all three release metrics. Release metrics also maintained strong positive intercorrelations at both concentrations.

## 4 Discussion

ARSs are a versatile drug delivery platform that enable *in situ*, controlled release using ultrasound. Potential applications include treatment of ischemic disease or bone defects via the localized delivery of pro-angiogenic or pro-osteogenic factors, respectively [7], [16], as well as treatment of post-operative infections using antibiotics [8]. Optimization of pharmacological strategies requires an understanding of how ARS and ultrasound properties impact overall performance. Rheological properties and drug release kinetics were dominated by the effects of fibrin concentration and acoustic pressure, respectively (Figs. 1, 3, 5). In previous studies, the impacts of acoustic pressure and fibrin concentration were dependent on formulation and ultrasound parameters [15], [18], [26], [27]. Thus, the presented factorial analysis identifies whether these plausible cross-effects alter the dominant control axes across the tested design space. This orthogonality carries design and practical implications. ARS stiffness is specified through formulation, which is not externally alterable after implantation. Conversely, acoustic pressure is externally alterable, thus enabling dynamic control of release kinetics. Here, varying acoustic pressure did not impact the modulus measured 12 days after ultrasound exposure. However, prior studies [25], [39] comparing the modulus before and ultrasound exposure demonstrate that ADV-generated bubbles increased the storage due to the strain-stiffening behavior of fibrin. Accordingly, the present rheological measurements are endpoint material properties that do not capture longitudinal changes in modulus post-ultrasound.

Bubble-derived metrics, which were also measured on day 12, reflect the complex behavior of dense bubbles within the ARS. Bubbles were generated by ADV, whose efficiency correlates with acoustic pressure in gels [15] and in liquid [40]. This was consistent with the primary dependence of bubble count on pressure (Figs. 1B, 4). However, bubble count also correlated, in a secondary manner, with fibrin concentration. One possible explanation for this association is reduced bubble coalescence at higher fibrin concentration, which can occur during ADV for neighboring bubbles in proximity as well as after ADV due to in-gassing and bubble growth. In the first scenario, bubble expansion during ADV was dampened at higher fibrin densities [41], which decreases the likelihood for coalescence. In the second scenario, bubble growth and coalescence were hindered in higher density gels [42], [43]. Bubble circularity and solidity exhibited moderated negative and reversed correlations with fibrin concentration, respectively. In prior studies, the irregularity in bubble shape correlated directly [44] and inversely [45] with hydrogel concentration.

Correlation patterns between bubble and release metrics shifted with pressure (Fig. 6). Cross-sectional and total surface bubble areas transitioned from non-significant or positive associations at low pressure to significant negative correlations at high pressure across all release metrics. Bubble count followed a similar pattern for daily and total release, whereas its correlation with fast release was not significant at high pressure. These pressure-stratified associations suggest a pressure-dependent change in the role bubbles play in release behavior. At both pressures, bubbles and drug release occurred as parallel outcomes of ADV. At high pressure, the negative correlations are consistent with a barrier-like or tortuosity-related effect of trapped bubbles on dextran transport. Gas-filled bubbles can reduce the aqueous volume available for solute transport and increase path complexity for hydrophilic macromolecules, consistent with prior observations that microbubbles alter dextran diffusivity in hydrogels and with effective-diffusivity models of obstructed media [46], [47], [48]. Prior work demonstrated that bubble growth is a primary driver of drug release from ARSs, with a multiplicative interaction between bubble growth and temperature governing release kinetics during the steady release phase (day 2 - 12) [26]. These findings are not contradictory. A possible explanation is that bubble growth exerts two competing effects: expanding bubbles mechanically compress ADV-generated, drug-laden fragments against the fibrin meshwork, increasing the contact area between payload and the surrounding aqueous phase and thereby promoting release; simultaneously, the growing gas phase occludes diffusion pathways, impeding solute transport. In the steady release phase, these opposing effects reach a dynamic equilibrium that sustains near-constant release. Additionally, median bubble area correlated negatively with release at low pressure, and exhibited larger areas than at high pressure. This pattern was consistent with less overall bubble formation, and hence release, at low pressure but a greater increase in individual bubble area due to low bubble density and dissolved gas partitioning among fewer bubbles [49]. Conversely, more extensive droplet vaporization at the higher acoustic pressure led to smaller bubbles due to the higher bubble density, and hence higher release.

Total surface bubble area and bubble count correlated positively with cross-sectional bubble area at low fibrin concentration (Fig. 7A). At high concentration, both bubble metrics exhibited higher values than at low concentration, but there were no significant correlations with cross-sectional area. The microenvironment of internal and surface bubbles were different, with the former experiencing constraint from the surrounding hydrogel matrix and neighboring bubbles, while the latter abutting the liquid interface. As a result, bubbles on the surface of the ARS are predicted to grow faster than internal bubbles [43]. The decoupling of surface bubble metrics (i.e., total area and count) from internal metrics at high cross-sectional areas suggests a limit to the number and area of bubbles that occupied the ARS surface. Interestingly, internal bubble characteristics clustered more closely with rheological properties rather than release metrics in the hierarchical analysis (Figs. 1A, 2A). This points to the impact of internal bubbles on macroscopic properties measured with bulk rheology.

The microstructure of a fibrin hydrogel is dependent on concentration, with fibril density and pore size correlating directly and indirectly with concentration, respectively. At high fibrin concentration, storage modulus correlated negatively with bubble count (Fig. 7A), whereas its associations with cross-sectional and total surface bubble areas were not significant. Similarly, in a prior study, the modulus of a crosslinked bovine serum albumin hydrogel decreased following entrapment of bubbles [50]. At low concentration, storage modulus was not significantly associated with bubble metrics. In parallel, release metrics were negatively correlated with storage modulus at high fibrin concentration but were not significantly correlated with storage modulus at low concentration (Fig. 7B). The hydrodynamic radius of 10 kDa dextran, the model drug used in this study, is 2.7 nm [51], which was considerable smaller than the pore diameters of 10 and 40 mg/mL fibrin hydrogels (i.e., approximately 1-10μm). However, molecular diffusivity decreases as fibrin concentration increases due to the increase in fiber density [52] and its impact on water types within the hydrogel [53]. These disparate trends highlight the importance of the initial fibrin concentration and associated network morphology on the mechanical properties and release kinetics of ARS.

In contrast to the dominant roles of concentration and pressure, scanning velocity and step size exhibited minimal main effects, accounting for less than 10% of total variance across all response variables (Fig. 1B). Velocity and step size are practically important since they affect the ultrasound exposure time. The number of acoustic pulses each droplet is exposed to (i.e., dwell time) is inversely correlated with velocity. Step size is inversely related to scan-line overlap and spatial coverage, thereby regulating the local density of ADV within the ARS. The weak main-effect behaviors may arise because velocity and step size primarily influence the initial spatial and temporal pattern of ADV, whereas the measured outcomes integrate subsequent longer-term processes like bubble growth and payload diffusion. The hypothesis regarding step size is supported by prior studies where release correlated inversely with step size within the first 10 h of ADV, but then became less significant or non-significant over 7-10 days [18], [26]. The observed negative correlation between release metrics and step size (Fig. 5) confirms that proper ultrasound exposure is critical for release. Furthermore, the prevalence of high-order interactions involving velocity and step size (Figs. 1, 2) suggests that while they do not fundamentally determine ARS behavior, they do impact the pressure response under specific matrix constraints.

Collectively, these findings highlight the central role of the $2^{4}$ full factorial design in this study. The objective extended beyond merely testing whether acoustic pressure alters release or whether fibrin concentration impacts stiffness. Instead, simultaneous variation of acoustic pressure, scanning velocity, scanning step size, and fibrin concentration enabled quantification of their relative contributions within a shared process–structure–function design space. This framework identified acoustic pressure as the dominant axis for release and fibrin concentration as the dominant axis for mechanics, supporting largely orthogonal control of release kinetics and final matrix mechanics within the tested range. The analysis also indicated that bubble morphology is not a universal surrogate for release, as associations between bubble morphology, release, and mechanics depended strongly on pressure and concentration. Finally, the weak main effects paired with specific interaction patterns for scanning velocity and scanning step size indicated that these parameters functioned primarily as conditional modulators rather than primary drivers. Thus, the full factorial design extended the analysis beyond repeated pairwise significance testing by defining parameter hierarchy, interaction structure, and response coupling across the ARS design space.

The main transferable contribution of this work is an experimental and analytical strategy for mapping how process parameters regulate scaffold microarchitecture, rheological properties, and release. Quantitative outputs from this framework (e.g., model coefficients, response magnitudes, ADV activation ranges, bubble metrics, and release correlations) are system specific. Here, ARSs with C${}_{7}$F_16_ emulsion yielded persistent, stable bubbles post-ADV, which underpins the observed process–structure–release relationships. ARSs that produce similarly persistent bubbles after ADV (e.g., with emulsions containing C${}_{5}$F_12_ or C${}_{6}$F_14_) may follow comparable design logic, whereas formulations that recondense after insonation (e.g., with C${}_{8}$F_18_ emulsion) require independent characterization. Acoustic pressure must also be interpreted relative to the ADV threshold of the selected formulation and ultrasound frequency, because droplet composition, frequency, and transducer configuration can shift the activation window. The present relationships were measured in vitro. Transfer to an in vivo setting requires system-specific calibration of two coupled boundary conditions: the acoustic exposure defined by implant geometry and tissue anatomy (e.g., propagation path, attenuation, and focal distortion) as well as the physiological transport environment (e.g., interstitial fluid transport and scaffold remodeling). Therefore, conditions that substantially alter ADV, post-ADV bubble persistence, acoustic exposure, or transport boundaries require further system-specific investigation.

This study has several limitations that should be acknowledged. First, bubble metrics and rheological properties were measured at a single terminal timepoint, whereas drug release was measured daily. Accordingly, the correlations reported linking drug release with bubble morphology and rheological properties should not be interpreted as direct evidence of dynamic, longitudinal structure–function coupling or temporal causality. In addition, the barrier-like effect of bubbles on dextran transport was inferred from release–morphology correlations; diffusion coefficients and in situ dextran distributions were not directly measured. Second, the two-level factorial design efficiently identified main effects and interactions but cannot resolve nonlinear relationships or pinpoint threshold values within the tested parameter ranges. Third, the use of a model payload (fluorescent dextran), which has minimal fibrin affinity and is highly mobile within the matrix, necessitates validation with specific therapeutic molecules to account for diverse drug-matrix interactions that could significantly alter diffusion and binding behaviors [54]. Therapeutic payloads with stronger matrix interactions, including fibrin-binding proteins (e.g., growth factors and cytokines), introduce an additional regulatory layer defined by matrix affinity, binding-site availability, and binding–dissociation kinetics. However, our prior studies with basic fibroblast growth factor, which binds to fibrin via its heparin-binding domains, reveal that ADV-mediated release is still dominated by acoustic pressure and is slowed by the presence of bubbles [55].

## 5 Conclusion

This study utilized a $2^{4}$ full factorial design to evaluate the effects of acoustic pressure, scanning velocity, scanning step size, and fibrin concentration on release kinetics, bubble morphology, and rheological properties of ARSs. Acoustic pressure governed drug release kinetics and fibrin concentration determined matrix mechanics, with neither parameter significantly affecting the other’s primary outcome. Bubble count was dependent on pressure and fibrin concentration, consistent with trends of ADV efficiency and bubble coalescence. Bubbles and drug release were linked, though at high pressure, bubbles were associated with reduced dextran release. Mechanical properties and release kinetics exhibited diverging trends with fibrin concentration, thus highlighting the importance of initial matrix concentration. Ultimately, this quantitative framework moves ARS design beyond empirical endpoint comparison by identifying parameter priorities, separable control axes, and condition-dependent structure–function relationships. These findings help elucidate critical relationships between process parameters and response variables, thus supporting the rational design of personalized therapeutic strategies using ARSs.

## CRediT authorship contribution statement

**Haijun Xiao:** Writing – original draft, Visualization, Methodology, Investigation, Formal analysis, Conceptualization. **Jinye Xie:** Investigation. **Somnath Maji:** Writing – review & editing. **Mitra Aliabouzar:** Writing – review & editing. **Mario L. Fabiilli:** Writing – review & editing, Supervision, Resources, Methodology, Funding acquisition, Formal analysis.

## Declaration of competing interest

The authors declare the following financial interests/personal relationships which may be considered as potential competing interests: Mario Fabiilli reports financial support was provided by National Institutes of Health. If there are other authors, they declare that they have no known competing financial interests or personal relationships that could have appeared to influence the work reported in this paper.

## Appendix A Supplementary data

MMC S1

## References

[1] Hu H., Busa P., Zhao Y., Zhao C. (2024). Externally triggered drug delivery systems. Smart Mater. Med..

[2] Yeingst T.J., Arrizabalaga J.H., Hayes D.J. (2022). Ultrasound-induced drug release from stimuli-responsive hydrogels. Gels.

[3] Xiao H., Aliabouzar M., Fabiilli M.L. (2026). Ultrasound-responsive composite hydrogels: Design rules for spatiotemporally controlled drug delivery. J. Control. Release.

[4] Yamakawa R., Onoe H., Kurashina Y. (2025). Hydrogel carrier with bubble vibration enhancer for ultrasound-triggered drug release. Ultrason. Sonochemistry.

[5] Orita Y., Shimanuki S., Okada S., Nakamura K., Nakamura H., Kitamoto Y., Shimoyama Y., Kurashina Y. (2023). Acoustic-responsive carbon dioxide-loaded liposomes for efficient drug release. Ultrason. Sonochemistry.

[6] Fabiilli M.L., Wilson C.G., Padilla F., Martín-Saavedra F.M., Fowlkes J.B., Franceschi R.T. (2013). Acoustic droplet–hydrogel composites for spatial and temporal control of growth factor delivery and scaffold stiffness. Acta Biomater..

[7] Maji S., Aliabouzar M., Quesada C., Chiravuri A., Macpherson A., Pinch A., Kazyak K., Emara Z., Abeid B.A., Kent R.N., Midekssa F.S., Zhang M., Baker B.M., Franceschi R.T., Fabiilli M.L. (2025). Ultrasound-generated bubbles enhance osteogenic differentiation of mesenchymal stromal cells in composite collagen hydrogels. Bioact. Mater..

[8] Fam Z.G.N., Winslow A., Fabiilli M.L., Varghese S., Oeffinger B.E., Forsberg F., Hickok N.J., Delaney L.J. (2026). Controlled antibiotic release from emulsion-loaded alginate and fibrin hydrogels using ultrasound. J. Biomed. Mater. Res. Part A.

[9] Dorvashi M., Harrison O.J., Sultan H.H., Zhang G., Thanou M., Ghavami N., Tiberi G., Ghavami M., Harput S. (2024). A meta-analysis of the effect of ultrasound activation parameters on phase-change nanodroplets in imaging and therapy. Front. Acoust..

[10] Burgess M.T., Porter T.M. (2019). Control of acoustic cavitation for efficient sonoporation with phase-shift nanoemulsions. Ultrasound Med. Biol..

[11] Al Rifai N., Stone K., Bo B., Zhang B., Kasiviswanathan D., Redington A.N., Haworth K.J. (2025). Assessing the oxygen scavenging capacity and myocardial gas embolization risk of ultrasonically activated phase shift perfluorobutane droplets. J. Mater. Chem. B.

[12] Ramesh R., Thimonier C., Desgranges S., Faugeras V., Coulouvrat F., Laurent J., Marrelec G., Contino-Pépin C., Urbach W., Tribet C., Taulier N. (2023). Acoustic droplet vaporization of perfluorohexane emulsions induced by heterogeneous nucleation at an ultrasonic frequency of 1.1 MHz. Langmuir.

[13] Olsman M., Mühlenpfordt M., Olsen E.B., Torp S.H., Kotopoulis S., Rijcken C.J., Hu Q., Thewissen M., Snipstad S., De Lange Davies C. (2021). Acoustic cluster therapy (ACT®) enhances accumulation of polymeric micelles in the murine brain. J. Control. Release.

[14] Vezeridis A.M., De Gracia Lux C., Barnhill S.A., Kim S., Wu Z., Jin S., Lux J., Gianneschi N.C., Mattrey R.F. (2019). Fluorous-phase iron oxide nanoparticles as enhancers of acoustic droplet vaporization of perfluorocarbons with supra-physiologic boiling point. J. Control. Release.

[15] Aliabouzar M., Lu X., Kripfgans O.D., Fowlkes J.B., Fabiilli M.L. (2019). Acoustic droplet vaporization in acoustically responsive scaffolds: Effects of frequency of excitation, volume fraction and threshold determination method. Ultrasound Med. Biol..

[16] Jin H., Quesada C., Aliabouzar M., Kripfgans O.D., Franceschi R.T., Liu J., Putnam A.J., Fabiilli M.L. (2021). Release of basic fibroblast growth factor from acoustically-responsive scaffolds promotes therapeutic angiogenesis in the hind limb ischemia model. J. Control. Release.

[17] Aliabouzar M., Quesada C., Chan Z.Q., Fowlkes J.B., Franceschi R.T., Putnam A.J., Fabiilli M.L. (2023). Acoustic droplet vaporization for on-demand modulation of microporosity in smart hydrogels. Acta Biomater..

[18] Lu X., Dong X., Natla S., Kripfgans O.D., Fowlkes J.B., Wang X., Franceschi R., Putnam A.J., Fabiilli M.L. (2019). Parametric study of acoustic droplet vaporization thresholds and payload release from acoustically-responsive scaffolds. Ultrasound Med. Biol..

[19] Xu S., Chang N., Wang R., Liu X., Guo S., Wang S., Zong Y., Wan M. (2018). Acoustic droplet vaporization and inertial cavitation thresholds and efficiencies of nanodroplets emulsions inside the focused region using a dual-frequency ring focused ultrasound. Ultrason. Sonochemistry.

[20] Wu Q., Mannaris C., May J.P., Bau L., Polydorou A., Ferri S., Carugo D., Evans N.D., Stride E. (2021). Investigation of the acoustic vaporization threshold of lipid-coated perfluorobutane nanodroplets using both high-speed optical imaging and acoustic methods. Ultrasound Med. Biol..

[21] Lajoinie G., Segers T., Versluis M. (2021). High-frequency acoustic droplet vaporization is initiated by resonance. Phys. Rev. Lett..

[22] Fiorini S., Prasanna A., Shakya G., Cattaneo M., Supponen O. (2025). Positive pressure matters in acoustic droplet vaporization. Phys. Rev. Res..

[23] Shpak O., Verweij M., Vos H.J., De Jong N., Lohse D., Versluis M. (2014). Acoustic droplet vaporization is initiated by superharmonic focusing. Proc. Natl. Acad. Sci..

[24] Guédra M., Coulouvrat F. (2015). A model for acoustic vaporization of encapsulated droplets. J. Acoust. Soc. Am..

[25] Aliabouzar M., Davidson C.D., Wang W.Y., Kripfgans O.D., Franceschi R.T., Putnam A.J., Fowlkes J.B., Baker B.M., Fabiilli M.L. (2020). Spatiotemporal control of micromechanics and microstructure in acoustically-responsive scaffolds using acoustic droplet vaporization. Soft Matter.

[26] Xiao H., Aliabouzar M., Fabiilli M.L. (2024). Acoustically responsive scaffolds: Unraveling release kinetics and mechanisms for sustained, steady drug delivery. J. Control. Release.

[27] Xiao H., Maji S., Pinch A., Aliabouzar M., Fabiilli M.L. (2025). Janus acoustically responsive scaffolds for sequential drug release with phase-programmed steady and pulsatile kinetics. J. Colloid Interface Sci..

[28] Aliabouzar M., Kripfgans O.D., Wang W.Y., Baker B.M., Brian Fowlkes J., Fabiilli M.L. (2021). Stable and transient bubble formation in acoustically-responsive scaffolds by acoustic droplet vaporization: Theory and application in sequential release. Ultrason. Sonochemistry.

[29] Zhao Y., Feng Y., Wu L. (2025). Process, dynamics and bioeffects of acoustic droplet vaporization induced by dual-frequency focused ultrasound. Ultrason. Sonochemistry.

[30] Leonidakis K.A., Bhattacharya P., Patterson J., Vos B.E., Koenderink G.H., Vermant J., Lambrechts D., Roeffaers M., Van Oosterwyck H. (2017). Fibrin structural and diffusional analysis suggests that fibers are permeable to solute transport. Acta Biomater..

[31] Fuenteslópez C.V., Bahcevanci S., Patrulea V., Ye H. (2026). Fibrin scaffolds for angiogenesis in soft tissue models: A systematic review. Bioact. Mater..

[32] Li S., Dan X., Chen H., Li T., Liu B., Ju Y., Li Y., Lei L., Fan X. (2024). Developing fibrin-based biomaterials/scaffolds in tissue engineering. Bioact. Mater..

[33] Elhelf I.S., Albahar H., Shah U., Oto A., Cressman E., Almekkawy M. (2018). High intensity focused ultrasound: The fundamentals, clinical applications and research trends. Diagn. Interv. Imaging.

[34] Garvin K.A., Hocking D.C., Dalecki D. (2010). Controlling the spatial organization of cells and extracellular matrix proteins in engineered tissues using ultrasound standing wave fields. Ultrasound Med. Biol..

[35] Aliabouzar M., Jivani A., Lu X., Kripfgans O.D., Fowlkes J.B., Fabiilli M.L. (2020). Standing wave-assisted acoustic droplet vaporization for single and dual payload release in acoustically-responsive scaffolds. Ultrason. Sonochemistry.

[36] Pachitariu M., Rariden M., Stringer C. (2025).

[37] Hartig F. (2024).

[38] Benjamini Y., Hochberg Y. (1995). Controlling the false discovery rate: A practical and powerful approach to multiple testing. J. R. Stat. Soc. Ser. B Stat. Methodol..

[39] Humphries B.A., Aliabouzar M., Quesada C., Bevoor A., Ho K.K.Y., Farfel A., Buschhaus J.M., Rajendran S., Fabiilli M.L., Luker G.D. (2022). Ultrasound-induced mechanical compaction in acoustically responsive scaffolds promotes spatiotemporally modulated signaling in triple negative breast cancer. Adv. Heal. Mater..

[40] Mistry N., Dhawan R., Manu K.S., Shekhar H., Mercado-Shekhar K.P. (2026). Gold nanoparticle coating reduces acoustic pressure threshold for perfluorohexane nanodroplet vaporization: Potential mechanisms and therapy implications. IEEE Trans. NanoBioscience.

[41] Aliabouzar M., Kripfgans O.D., Estrada J.B., Brian Fowlkes J., Fabiilli M.L. (2022). Multi-time scale characterization of acoustic droplet vaporization and payload release of phase-shift emulsions using high-speed microscopy. Ultrason. Sonochemistry.

[42] Aviño F., Matheson A.B., Adams D.J., Clegg P.S. (2017). Stabilizing bubble and droplet interfaces using dipeptide hydrogels. Org. Biomol. Chem..

[43] Ando K., Shirota E. (2019). Quasistatic growth of bubbles in a gelatin gel under dissolved-gas supersaturation. Phys. Fluids.

[44] Haudin F., Noblin X., Bouret Y., Argentina M., Raufaste C. (2016). Bubble dynamics inside an outgassing hydrogel confined in a hele-shaw cell. Phys. Rev. E.

[45] Yang J., Cramer H.C., Bremer E.C., Buyukozturk S., Yin Y., Franck C. (2022). Mechanical characterization of agarose hydrogels and their inherent dynamic instabilities at ballistic to ultra-high strain-rates via inertial microcavitation. Extrem. Mech. Lett..

[46] Lima E.G., Durney K.M., Sirsi S.R., Nover A.B., Ateshian G.A., Borden M.A., Hung C.T. (2012). Microbubbles as biocompatible porogens for hydrogel scaffolds. Acta Biomater..

[47] Axpe E., Chan D., Offeddu G.S., Chang Y., Merida D., Hernandez H.L., Appel E.A. (2019). A multiscale model for solute diffusion in hydrogels. Macromolecules.

[48] Di Felice R., Drioli E., Giorno L. (2016). Encyclopedia of Membranes.

[49] Daneshi M., Frigaard I. (2023). Growth and stability of bubbles in a yield stress fluid. J. Fluid Mech..

[50] Brown C.P., Hughes M.D.G., Mahmoudi N., Brockwell D.J., Coletta P.L., Peyman S., Evans S.D., Dougan L. (2023). Structural and mechanical properties of folded protein hydrogels with embedded microbubbles. Biomater. Sci..

[51] Goins A.B., Sanabria H., Waxham M.N. (2008). Macromolecular crowding and size effects on probe microviscosity. Biophys. J..

[52] Ghajar C.M., Chen X., Harris J.W., Suresh V., Hughes C.C., Jeon N.L., Putnam A.J., George S.C. (2008). The effect of matrix density on the regulation of 3-D capillary morphogenesis. Biophys. J..

[53] Dromel P.C., Singh D., Christoff-Tempesta T., Martheswaran T., Alexander-Katz A., Spector M., Young M. (2021). Controlling growth factor diffusion by modulating water content in injectable hydrogels. Tissue Eng. Part A.

[54] Martino M.M., Briquez P.S., Ranga A., Lutolf M.P., Hubbell J.A. (2013). Heparin-binding domain of fibrin(ogen) binds growth factors and promotes tissue repair when incorporated within a synthetic matrix. Proc. Natl. Acad. Sci..

[55] Dong X., Lu X., Kingston K., Brewer E., Juliar B.A., Kripfgans O.D., Fowlkes J.B., Franceschi R.T., Putnam A.J., Liu Z., Fabiilli M.L. (2019). Controlled delivery of basic fibroblast growth factor (bFGF) using acoustic droplet vaporization stimulates endothelial network formation. Acta Biomater..

