# Supplementary material for: From microbubbles to macro-control: Linking ultrasound-induced microarchitecture to precise drug release kinetics from acoustically responsive scaffolds
Source: Ultrason Sonochem. 2026 Jul 22;132:107965. doi: 10.1016/j.ultsonch.2026.107965 (PMC13427563; doi:10.1016/j.ultsonch.2026.107965)
Supplement: MMC S1 [file mmc1.pdf]

From Microbubbles to Macro-Control: Linking Ultrasound-Induced Microarchitecture to Precise Drug Release Kinetics from Acoustically Responsive Scaffolds

Haijun Xiao[1], Jinye Xie[2], Somnath Maji[1], Mitra Aliabouzar[1,3], Mario L. Fabiilli[1,2,4,*]

Supplementary Materials

2026-07-07

***Address:*.**

[1] *Department of Radiology, University of Michigan, Ann Arbor, MI, USA, 48109.*

[2] *Department of Biomedical Engineering, University of Michigan, Ann Arbor, MI, USA, 48109.*

[3] *Department of Mechanical Engineering, University of Michigan, Ann Arbor, MI USA, 48109.*

[4] *Applied Physics Program, University of Michigan, Ann Arbor, MI, USA, 48109.*

***Contact Information:*.**

Dr. Haijun Xiao, Ph.D.

Email: haijun.xiao@urz.one.

Jinye Xie.

Email: jscxie@umich.edu.

Dr. Somnath Maji, Ph.D.

Email: smaji@umich.edu.

Dr. Mitra Aliabouzar, Ph.D.

Email: aliabouz@umich.edu.

* *Dr. Mario L. Fabiilli, Ph.D.*

Email: mfabiill@umich.edu

Table of Contents 2

1 Methods 3
1.1 Experimental Design . . . 3
1.2 Data Acquisition . . . 3
1.3 Bubble Segmentation and Morphological Analysis . . . 4
1.3.1 Cross-Sectional Bubble Area Quantification . . . 4
1.4 Statistical Modeling Framework . . . 4
1.4.1 Statistical Software . . . 4
1.4.2 Data Preprocessing . . . 5
1.4.3 Model Specification . . . 5
1.4.4 Model Selection . . . 5
1.4.5 Residual Diagnostics and Inference Strategy . . . 6
1.4.6 Modeled Response Variables . . . 6
1.4.7 Multiple Comparison Adjustment . . . 6
1.5 Cross-Metric Effect Analysis . . . 7
1.5.1 Process Parameter Reliability and Variance Contribution . . . 7
1.5.2 Standardized Coefficients and Directional Stability . . . 7
1.5.3 Process Parameter Prioritization Framework . . . 8
1.5.4 Response Correlation Analysis . . . 9

2 Results 10

References 18

1 Methods

1.1 Experimental Design

A 2^4 full factorial design was employed to systematically evaluate the effects of four process parameters on scaffold properties: peak negative pressure, scanning velocity, scanning step size, and fibrin concentration (Table S1). Each parameter was tested at two levels, producing 16 unique parameter combinations. All combinations were performed in triplicate, yielding a total of 48 independent samples.

Table S1: Full 2^4 factorial experimental design and condition-label map.

Sonication	Run	Pressure (MPa)	Velocity (mm/s)	Step Size (mm)	Concentration (mg/mL)
A	1	2.9	3	1.3	10
	2	2.9	3	1.3	40
B	3	3.9	3	1.3	10
	4	3.9	3	1.3	40
C	5	2.9	6	1.3	10
	6	2.9	6	1.3	40
D	7	3.9	6	1.3	10
	8	3.9	6	1.3	40
E	9	2.9	3	2.6	10
	10	2.9	3	2.6	40
F	11	3.9	3	2.6	10
	12	3.9	3	2.6	40
G	13	2.9	6	2.6	10
	14	2.9	6	2.6	40
H	15	3.9	6	2.6	10
	16	3.9	6	2.6	40

Note: Sonication labels A–H denote the eight ultrasound-scanning conditions defined by acoustic pressure, scanning velocity, and step size; each sonication label was evaluated at both fibrin concentrations, yielding 16 run-level factorial conditions.

1.2 Data Acquisition

All measurements followed a standardized timeline relative to ultrasound treatment (t_0). Table S2 summarizes the acquisition schedule and instrumentation for each response category.

Table S2: Response variables measured in the 2^4 full factorial experiment.

Category	Variables	Time Point	Method
**Drug Release**	Fast release, average daily release, total release	$t_0 - t_{12}$	Daily medium exchange, fluorescence spectrophotometry
**Cross-Sectional Bubble**	Cross-sectional bubble area	t_{12}	B-mode ultrasound imaging
**Surface Bubble**	Count, median area, surface total area, circularity, solidity	t_{12}	Brightfield imaging, instance segmentation (Cellpose)
**Rheology**	Storage modulus (G'), loss modulus (G''), loss tangent ($\tan\delta$)	t_{12}	Oscillatory amplitude sweep rheometry

1.3 Bubble Segmentation and Morphological Analysis

1.3.1 Cross-Sectional Bubble Area Quantification

Within each B-mode image, a rectangular region of interest (ROI) was defined to exclude BioFlex wall reflections. Bubble cross-sectional area was quantified using the Reflector Shape Reconstruction and Projection (RSRP) algorithm. The presence of large bubbles within the gel created acoustically shadowed regions distal to the ultrasound imaging probe. To account for these anechoic regions, cross-sectional area was therefore estimated by vertical projection of the detected upper surface boundary.

The algorithm operated in three stages. First, reflector reconstruction identified the bubble's upper surface. Intensity thresholding at 130 isolated bright reflections from the background. Morphological opening (2 iterations) removed noise structures smaller than approximately 4 pixels, while subsequent closing bridged discontinuities caused by specular reflections at oblique surface angles. Second, vertical projection generated the cross-sectional mask. For each image column, the algorithm identified the uppermost detected reflector pixel and designated all pixels from that point to the bottom of the ROI as bubble-affected area. Linear interpolation bridged gaps narrower than 15 pixels when valid detections existed on both sides. Third, bubble-affected pixels were summed and converted to physical area (32.69 μm/pixel, mm^2) using the image scale calibration.

The algorithm was implemented in Fiji and applied to all acquired images with consistent parameters.

1.4 Statistical Modeling Framework

1.4.1 Statistical Software

All analyses were performed in R (4.5.2)[1]. Data manipulation and transformation used the **tidyverse** ecosystem[2]. Statistical models were fitted using base R and the **car** package[3]. Model diagnostics were conducted with **DHARMa**[4] and **performance**[5]. Robust standard errors were computed via **sandwich**[6–8], and likelihood ratio tests were performed with **lmtest**[9]. Cross-validation was implemented using **caret**[10].

Visualization was built with **ggplot2**[11] and **scales**[12]. Multi-panel figures were assembled with **patchwork**[13]. Non-overlapping labels were placed using **ggrepel**[14], dendrograms rendered with **ggdendro**[15], and paired

correlation matrices generated with **GGally**[16]. SVG output was rendered via **rsvg**[17].

1.4.2 Data Preprocessing

All four process parameters were centered by subtracting their respective grand means prior to model fitting. Centering ensured that intercept estimates represented predicted responses at average process conditions, reduced multicollinearity between main effects and their derived interaction terms, and permitted interpretation of each main effect as the expected change in response per unit increase in that parameter while all remaining parameters were held at their mean levels.

1.4.3 Model Specification

For each response variable, a full factorial linear model was fitted containing all main effects and interaction terms up to fourth order (Equation 1):

$$y = \beta_0 + \sum_i \beta_i x_i + \sum_{i<j} \beta_{ij} x_i x_j + \sum_{i<j<k} \beta_{ijk} x_i x_j x_k + \beta_{1234} x_1 x_2 x_3 x_4 + \varepsilon \quad (1)$$

where x_1, x_2, x_3, and x_4 denote the centered values of acoustic pressure, scanning velocity, step size, and fibrin concentration, respectively. In this two-level factorial design, each regression coefficient β_i represents half the effect of the corresponding factor, and the full effect equals $2\beta_i$.

1.4.4 Model Selection

Three candidate models were evaluated for each response variable. The first was the full factorial model containing all 15 terms (four main effects, six two-factor interactions, four three-factor interactions, and one four-factor interaction). The second was a stepwise-reduced model obtained through bidirectional Akaike information criterion (AIC) minimization. The third was a DHARMa-optimized model constructed by retaining all four main effects as mandatory terms and adding only those higher-order terms identified as statistically significant ($p < 0.05$) by either Type III analysis of variance or robust Wald testing, depending on the outcome of preliminary residual diagnostics.

Selection among the three candidates employed a composite quality score. The base score was defined as $R^2_{\text{adj}} \times 100$. Penalty terms were applied for failures of DHARMa diagnostic tests (uniformity: 25 points; dispersion: 30 points; outliers: 15 points) and for model complexity ($\log(p + 1) \times 8$, where p denotes the number of estimated parameters). Models passing all DHARMa tests at the preliminary screening stage (2500 simulations) were prioritized; among passing candidates, the highest composite score determined selection. When no candidate

passed preliminary screening, the model with the highest composite score was selected and subjected to enhanced final diagnostics. Likelihood ratio tests confirmed that simplification did not significantly reduce explanatory power relative to the full model.

1.4.5 Residual Diagnostics and Inference Strategy

Model assumptions were evaluated using the DHARMa package, which assesses distributional adequacy through simulation-based scaled residuals. For each selected model, 5000 simulations generated reference distributions against which three core diagnostic tests were applied: (i) a Kolmogorov–Smirnov uniformity test evaluating whether scaled residuals followed the expected uniform distribution, (ii) a dispersion test comparing observed residual variance against simulated expectations, and (iii) an outlier test identifying observations with extreme residual values. The outcome of these tests determined the inferential strategy: models passing all three tests ($p > 0.05$) were analyzed using standard Type III ANOVA; models failing one or more tests were analyzed using heteroscedasticity-consistent (HC3) robust Type III ANOVA to ensure valid inference under potential violations of homoscedasticity.

Predictive accuracy was assessed by 10-fold cross-validation with a fixed random seed to ensure reproducibility, yielding root mean square error (CV RMSE) estimates. Multicollinearity among model terms was evaluated using variance inflation factors (VIF), with values below 5 considered acceptable.

1.4.6 Modeled Response Variables

Twelve response variables spanning three categories were modeled: drug release kinetics (fast release, average daily release, total release), rheological properties (storage modulus, loss modulus, loss tangent), bubble morphology (cross-sectional bubble area, surface bubble count, circularity, solidity, median bubble area, surface total bubble area).

1.4.7 Multiple Comparison Adjustment

To control the false discovery rate (FDR) arising from simultaneous testing of multiple effects across multiple response variables, all p-values were adjusted using the Benjamini–Hochberg procedure[18]. An adjusted threshold of $p_{\mathrm{FDR}} < 0.05$ was applied for all subsequent effect prioritization and significance assessments.

1.5 Cross-Metric Effect Analysis

1.5.1 Process Parameter Reliability and Variance Contribution

To compare effect importance across response variables, two complementary metrics were computed for each effect–response combination. The absolute t value ($|t|$) quantified statistical signal strength, defined as the ratio of the estimated coefficient to its standard error. Variance contribution (VC%) quantified each effect's relative share of model-explained variance, calculated as (Equation 2):

$$\mathrm{VC}_j\% = \frac{SS_j}{\sum_k SS_k} \times 100\% \tag{2}$$

where SS_j denotes the Type III sum of squares for the j-th effect and the summation extends over all model effects excluding the intercept and residuals. This normalization enabled cross-response comparison by expressing each effect's importance as a percentage of total model-explained variance rather than in response-specific units.

Both metrics were arranged into effect-by-response matrices. Hierarchical clustering was applied to rows (responses) and columns (effects) using correlation-based distance ($1 - $ Pearson r) and Ward's minimum variance method (Ward.D2), grouping responses with similar sensitivity profiles and effects with similar influence patterns regardless of absolute magnitude. Black borders on heatmap cells denoted FDR-adjusted significance ($p_{\mathrm{FDR}} < 0.05$).

1.5.2 Standardized Coefficients and Directional Stability

To enable magnitude comparison across effects measured on different scales, standardized coefficients (β^*) were computed for each effect–response pair (Equation 3):

$$\beta_j^* = \hat{\beta}_j \times \frac{SD_{X_j}}{SD_Y} \tag{3}$$

where $\hat{\beta}_j$ is the unstandardized regression coefficient, SD_{X_j} is the standard deviation of the predictor term (for interaction terms, the product of constituent variable standard deviations), and SD_Y is the standard deviation of the response variable computed from the original (uncentered) data. The resulting β^* represents the expected change in the response, in standard deviation units, per one standard deviation change in the predictor.

Directional consistency was assessed for all FDR-significant effects by evaluating conditional marginal effects across the design space. For each significant effect, the marginal effect (ME) was defined as (Equation 4):

$$\text{ME(target)} = \beta_{\text{target}} + \sum_k \beta_{\text{target}:M_k} \times M_k \tag{4}$$

where M_k denotes the moderator variables appearing in higher-order interaction terms that contain the target effect, and the summation includes only those higher-order terms that were themselves FDR-significant. The marginal effect was evaluated at all corner points of the moderator variable ranges (the extremes of the centered design space), yielding a distribution of conditional effects with associated confidence intervals based on the t distribution. Each effect was classified into one of three stability categories based on the signs and significance of marginal effects across all corner-point conditions: *robust* (consistent direction and significance across all conditions), *moderated* (consistent sign but variable magnitude, with some conditions losing significance), or *reversed* (sign change across conditions, with at least one significantly positive and one significantly negative marginal effect). Verification of marginal effect calculations was performed by comparing analytical results against manual computation and numerical differentiation, with maximum discrepancies confirmed to be below 10^{-10}.

1.5.3 Process Parameter Prioritization Framework

A quadrant framework was constructed to integrate statistical reliability with practical importance for each effect–response combination. The horizontal axis represented FDR-adjusted statistical significance, plotted as $-\log_{10}(p_{\text{FDR}})$, with a vertical threshold at $p_{\text{FDR}} = 0.05$ (corresponding to $-\log_{10}(0.05) \approx 1.30$). The vertical axis represented variance contribution (VC%) on a logarithmic scale ($\log_{10}(\text{VC\%} + 1)$), with a horizontal threshold at VC% = 10%, corresponding to approximately 1.5 times the expected contribution under uniform distribution across the 15 effects in the full factorial model.

Effects were classified into four quadrants following Cartesian convention: Quadrant I (upper right: statistically significant and high variance contribution), Quadrant II (upper left: high variance contribution but not statistically significant), Quadrant III (lower left: neither significant nor high variance contribution), and Quadrant IV (lower right: statistically significant but low variance contribution). Directional stability classifications were encoded as point shapes on the quadrant plots, enabling simultaneous assessment of reliability, importance, and directional consistency.

The framework was applied globally across all twelve response variables and separately for each of three response categories: rheological properties, bubble morphology, and drug release kinetics. Per-response faceted views used shared axis scales within each category to facilitate direct comparison.

1.5.4 Response Correlation Analysis

Pairwise Pearson correlations among response variables were computed and stratified by acoustic pressure (low vs. high) and fibrin concentration (low vs. high) to identify condition-dependent relationships. For each variable pair, correlation coefficients were computed within each stratum, and the significance of between-stratum differences was evaluated using Fisher's z transformation (Equation 5):

$$z = \frac{z_1 - z_2}{\sqrt{\frac{1}{n_1-3} + \frac{1}{n_2-3}}} \tag{5}$$

where z_1 and z_2 are the Fisher-transformed correlations from the two strata and n_1 and n_2 are the respective sample sizes ($n = 24$ per stratum). Correlation patterns were classified as stable (consistent across strata), reversed (sign change between strata), decoupled (significant in one stratum, absent in the other), or emergent (absent overall, significant within strata). Paired correlation matrices with scatter plots, regression lines, and density distributions were generated separately for pressure-stratified and concentration-stratified analyses.

2 Results

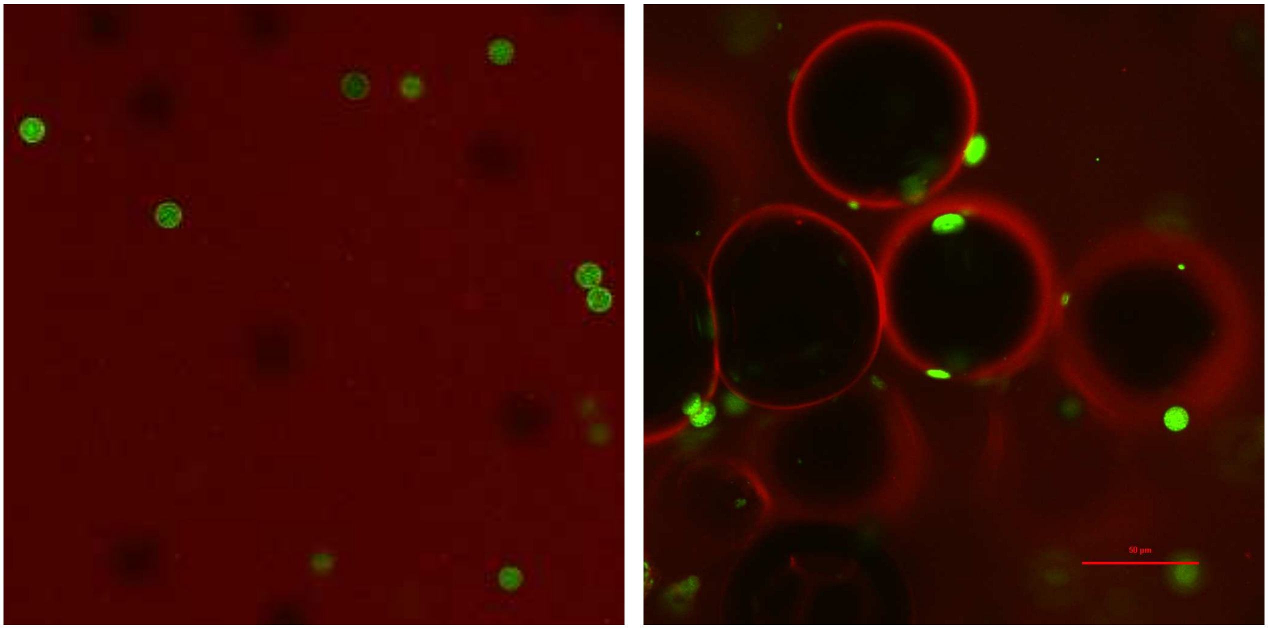

Figure S1: Representative confocal fluorescence micrographs of ARSs before and after ultrasound exposure. Left: ARS before ultrasound exposure. Right: ARS after ultrasound exposure. Green fluorescence indicates Alexa Fluor 488-labeled dextran; red fluorescence indicates Alexa Fluor 647-labeled fibrin. Dark circular regions with bright red boundaries in the post-ultrasound panel indicate bubble cross-sections.

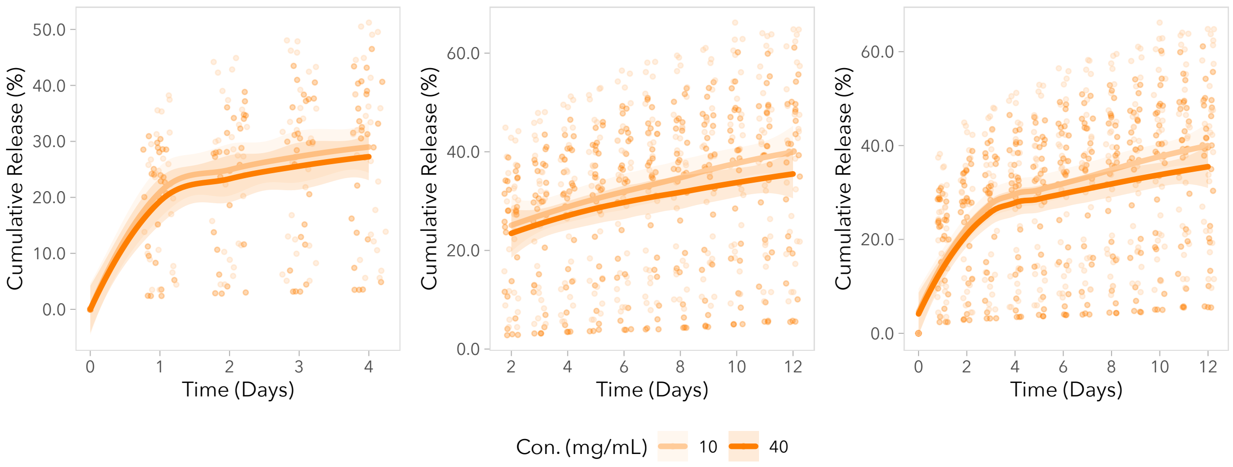

Figure S2: Cumulative drug release profiles grouped by fibrin concentration. Left: fast release phase (days 0-1). Center: sustained release phase (days 2–12). Right: complete release profile (days 0–12). Individual data points represent triplicate measurements per experimental condition. Points at day 0 are plotted without jitter while subsequent timepoints are horizontally jittered (± 0.25 days) for visual clarity. Lines represent LOESS smoothing fits per concentration level; shaded bands indicate 95% confidence intervals. $n = 24$ per concentration level.

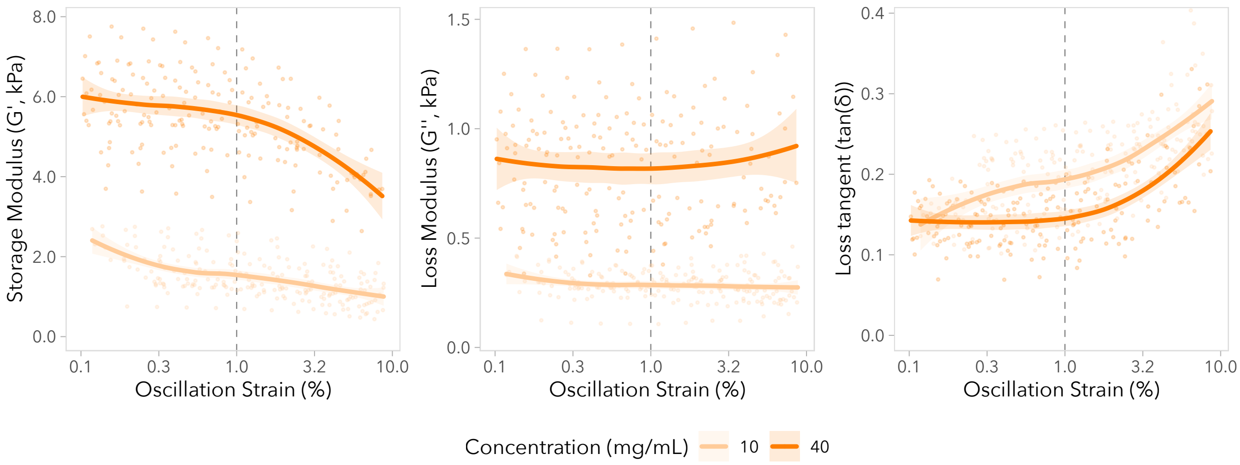

Figure S3: Amplitude sweep rheological characterization grouped by fibrin concentration. Left: storage modulus (G'). Center: loss modulus (G''). Right: loss tangent ($\tan\delta = G''/G'$). Oscillation strain is plotted on a logarithmic scale; the vertical dashed line indicates 1% strain, corresponding to the measurement point used for statistical modeling. In the loss tangent panel, the horizontal red line marks $\tan\delta = 1$, above which viscous behavior dominates over elastic behavior. Lines represent LOESS fits; shaded bands indicate 95% confidence intervals. $n = 24$ per concentration level.

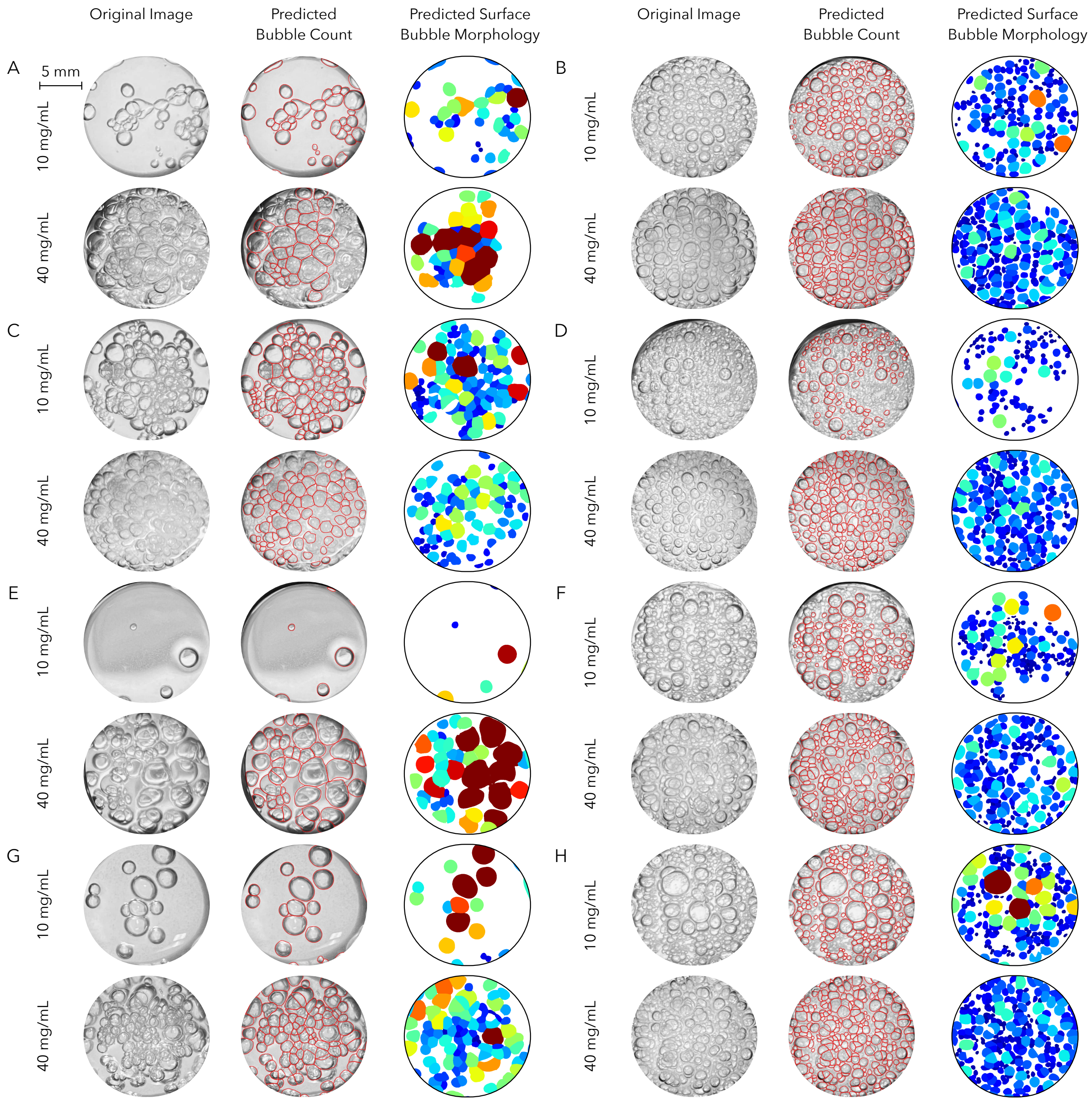

Figure S4: Representative surface bubble segmentation results across all experimental conditions. Panels A–H correspond to the eight sonication conditions defined in Table S1, each represented at both fibrin concentration levels (10 and 40 mg/mL). For each representative sample, three panels are shown: the original brightfield image (left), the segmentation overlay with detected bubble contours outlined in red on the original image (center), and the predicted bubble morphology mask with individual bubbles colored by area using a continuous color scale (right). One of three replicate wells is shown per condition.

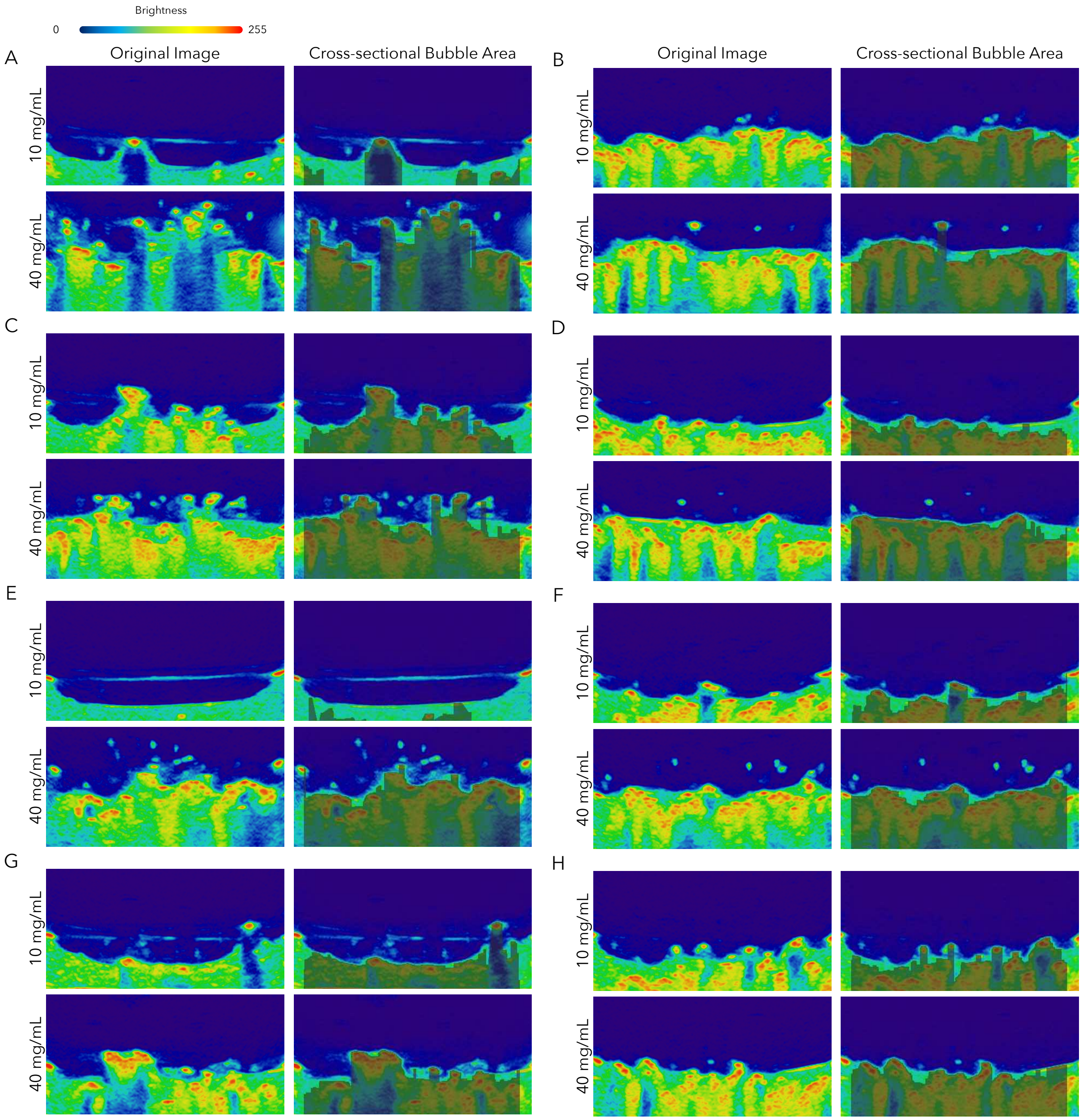

Figure S5: Representative B-mode ultrasound cross-sectional images across all experimental conditions. Panels A–H correspond to the eight sonication conditions defined in Table S1, each represented at both fibrin concentration levels (10 and 40 mg/mL). For each representative sample, two panels are shown: the original B-mode ultrasound image (left) and the same image overlaid with the detected cross-sectional bubble area mask (right). One of three replicate wells is shown per condition.

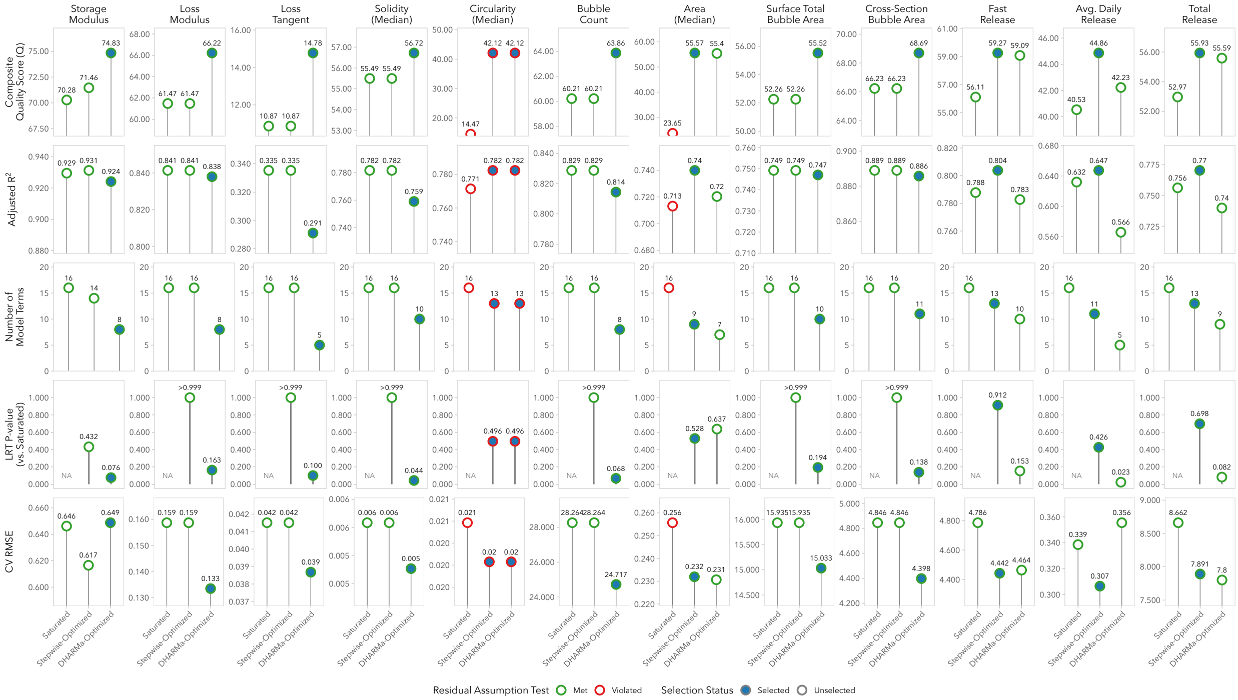

Figure S6: Comprehensive evaluation and selection framework for candidate models across all response variables. The performance of three candidate models (saturated, stepwise-optimized, and DHARMa-optimized) is compared across five metrics for each response variable. (A) Composite quality score (Q) integrating model fit, complexity, and diagnostic validity, serving as the primary selection criterion. (B) Goodness-of-fit, measured by R^2_{adj}. (C) Model complexity, indicated by the number of model terms. (D) Parsimony validation: p-values from likelihood ratio tests comparing each reduced model against the saturated model (NA for the saturated model itself). (E) Predictive accuracy, assessed by 10-fold cross-validated root mean square error (CV RMSE). Point border color indicates DHARMa residual diagnostic status (green: assumptions met; red: assumptions violated). Point fill indicates selection status (blue-filled: selected; open: not selected). See Methods for composite score definition and selection criteria.

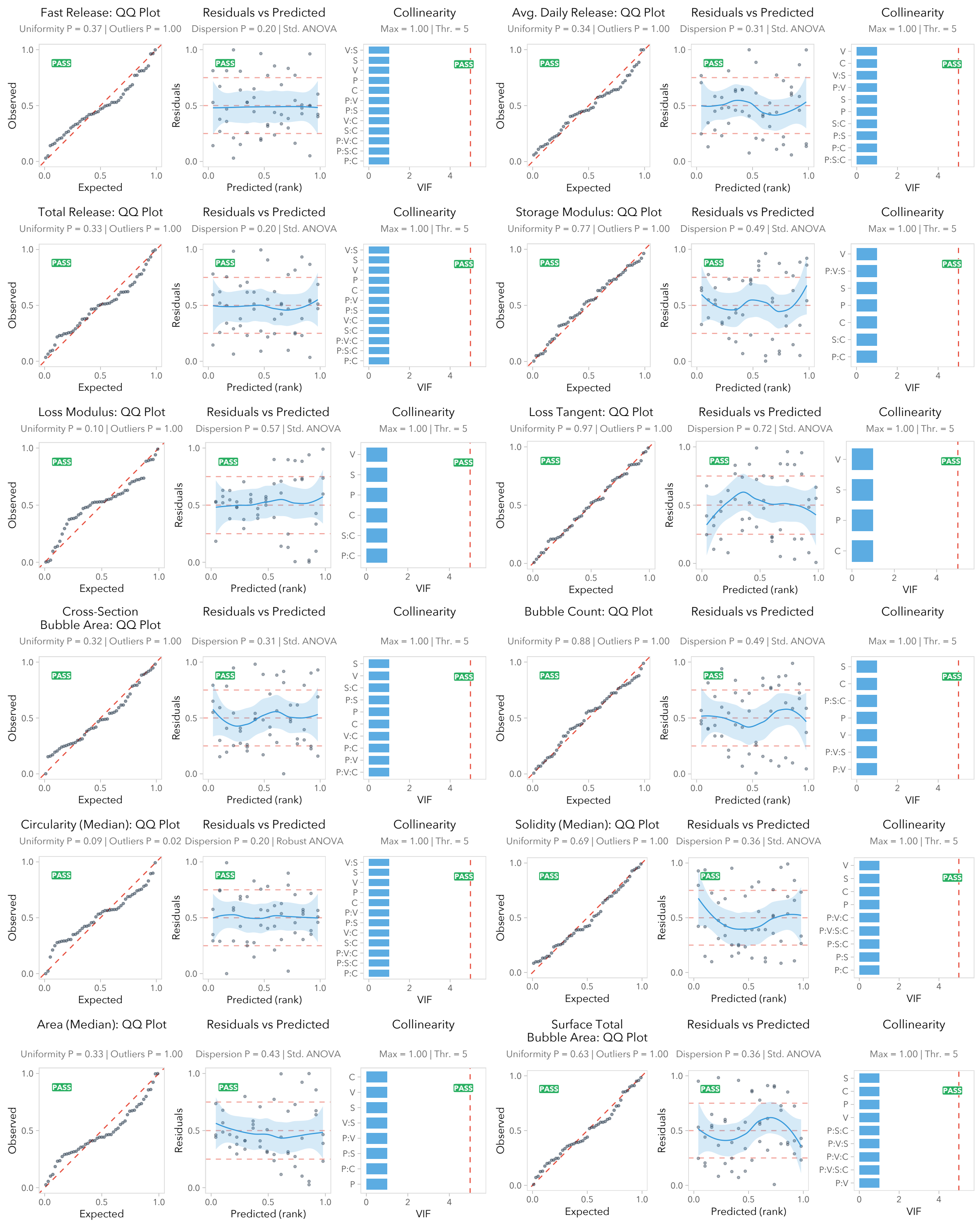

Figure S7: DHARMa residual diagnostics and collinearity assessment for all twelve selected models. For each model, three diagnostic panels are shown: quantile-quantile plot of simulation-based scaled residuals for uniformity and outlier detection, scaled residuals versus rank-transformed predicted values for dispersion assessment, and variance inflation factors (VIF) for multicollinearity evaluation. Green PASS or red FAIL labels indicate whether each criterion was met ($p > 0.05$ for DHARMa tests; VIF < 5 for collinearity). Models failing one or more tests were analyzed using heteroscedasticity-consistent (HC3) robust Type III ANOVA. Diagnostics based on 5000 simulations per model. Abbreviations: P = Pressure, V = Velocity, C = Concentration, S = Step size.

To further assess the low adjusted R^2 of the loss-tangent model, triplicate-level repeatability was summarized for each rheological response across the 16 factorial conditions. Within-condition coefficients of variation (CVs) were calculated separately for storage modulus, loss modulus, and loss tangent, and the interquartile range (IQR) was used to summarize the dispersion of CVs across conditions.

Table S3: Triplicate-level repeatability of rheological responses across factorial conditions.

Response	Conditions	Median within-condition CV (%)	IQR of within-condition CV (%)	Mean within-condition CV (%)
Storage modulus	16	16.3	18.9	17.1
Loss modulus	16	13.5	18.9	17.1
Loss tangent	16	18.0	14.0	20.1

The loss-tangent main-effect model was also compared against the saturated factorial model. Adding all higher-order process interactions increased adjusted R^2 from 0.291 to 0.335 but did not significantly improve fit ($p = 0.291$). Pearson correlations between loss-tangent residuals and measured release, bubble, and rheological variables yielded no FDR-significant associations.

References

1. R Core Team. *R: A Language and Environment for Statistical Computing*. (R Foundation for Statistical Computing, Vienna, Austria, 2025).
2. Wickham, H. *et al.* Welcome to the tidyverse. *Journal of Open Source Software* **4**, 1686 (2019).
3. Fox, J. & Weisberg, S. *An R Companion to Applied Regression*. (Sage, Thousand Oaks CA, 2019).
4. Hartig, F. *DHARMa: Residual Diagnostics for Hierarchical (Multi-Level / Mixed) Regression Models*. (2024). doi:10.32614/CRAN.package.DHARMa.
5. Lüdecke, D., Ben-Shachar, M. S., Patil, I., Waggoner, P. & Makowski, D. performance: An R package for assessment, comparison and testing of statistical models. *Journal of Open Source Software* **6**, 3139 (2021).
6. Zeileis, A. Econometric computing with HC and HAC covariance matrix estimators. *Journal of Statistical Software* **11**, 1–17 (2004).
7. Zeileis, A. Object-oriented computation of sandwich estimators. *Journal of Statistical Software* **16**, 1–16 (2006).
8. Zeileis, A., Köll, S. & Graham, N. Various versatile variances: An object-oriented implementation of clustered covariances in R. *Journal of Statistical Software* **95**, 1–36 (2020).
9. Zeileis, A. & Hothorn, T. Diagnostic checking in regression relationships. *R News* **2**, 7–10 (2002).
10. Kuhn & Max. Building predictive models in R using the caret package. *Journal of Statistical Software* **28**, 1–26 (2008).
11. Wickham, H. *Ggplot2: Elegant Graphics for Data Analysis*. (Springer-Verlag New York, 2016).
12. Wickham, H., Pedersen, T. L. & Seidel, D. *Scales: Scale Functions for Visualization*. (2025). doi:10.32614/CRAN.package.scales.
13. Pedersen, T. L. *Patchwork: The Composer of Plots*. (2025). doi:10.32614/CRAN.package.patchwork.
14. Slowikowski, K. *Ggrepel: Automatically Position Non-Overlapping Text Labels with 'Ggplot2'*. (2024). doi:10.32614/CRAN.package.ggrepel.
15. de Vries, A. & Ripley, B. D. *Ggdendro: Create Dendrograms and Tree Diagrams Using 'Ggplot2'*. (2024). doi:10.32614/CRAN.package.ggdendro.
16. Schloerke, B. *et al. GGally: Extension to 'Ggplot2'*. (2025). doi:10.32614/CRAN.package.GGally.
17. Ooms, J. *Rsvg: Render SVG Images into PDF, PNG, (Encapsulated) PostScript, or Bitmap Arrays*. (2025). doi:10.32614/CRAN.package.rsvg.

18. Benjamini, Y. & Hochberg, Y. Controlling the false discovery rate: A practical and powerful approach to multiple testing. *Journal of the Royal Statistical Society Series B (Statistical Methodology)* 57, 289–300 (1995).
